# Meta-Prediction of MTHFR Gene Polymorphism and Air Pollution on the Risks of Congenital Heart Defects Worldwide: A Transgenerational Analysis

**DOI:** 10.3390/ijerph15081660

**Published:** 2018-08-05

**Authors:** Hsiao-Ling Yang, Ya-Ling Yang, Chong Ho Yu, S. Pamela K. Shiao

**Affiliations:** 1 School of Nursing, College of Medicine, National Taiwan University, Taipei 10051, Taiwan; slyang@ntu.edu.tw (H.-L.Y.); ylyang@ntu.edu.tw (Y.-L.Y.); 2 Department of Psychology, Azusa Pacific University, Azusa, CA 91702, USA; cyu@apu.edu; 3 College of Nursing, Augusta University, Augusta, GA 30912, USA

**Keywords:** *methylenetetrahydrofolate reductase*, polymorphism, heart defect, congenital, air pollution, meta-predictive analysis

## Abstract

Congenital heart disease (CHD) is the leading cause of death in children, and is affected by genetic and environmental factors. To investigate the association of air pollution with *methylene-tetrahydrofolate reductase* (*MTHFR*) polymorphisms and the risk of CHD, we included 58 study groups of children and parents, with 12,347 cases and 18,106 controls worldwide. Both *MTHFR* C677T (rs 1801133) and A1298C (rs 1801131) gene polymorphisms were risks for CHD in children with transgenerational effects from their parents. Countries with greater risks of CHD with a pooled risk ratio (RR) > 2 from *MTHFR* 677 polymorphisms included Germany, Portugal, China, and Egypt for children; and Brazil, Puerto Rico, Mexico, China, and Egypt for mothers. Whereas, countries with greater risk of CHD with RR > 2 from *MTHFR* 1298 polymorphisms included Taiwan, Turkey, and Egypt for children; and Brazil, China, and Egypt for mothers. Additionally, meta-prediction analysis revealed that the percentages of *MTHFR* 677TT and TT plus CT polymorphisms together were increased in countries with higher levels of air pollution, with a trend of increased CHD risks with higher levels of air pollution for children (*p* = 0.07). Our findings may have significant implications for inflammatory pathways in association with *MTHFR* polymorphisms and future intervention studies to correct for folate-related enzyme deficits resulted from *MTHFR* polymorphisms to prevent CHDs for future generations.

## 1. Introduction

Congenital heart disease (CHD) is the most common congenital abnormity and causes most mortality in children worldwide [1,2]. With advanced surgical procedures, 90% of CHD cases could grow up into adulthood [3]. Many causes of CHD have been noted including chromosomal anomalies or gene mutations; maternal pregnancy complications including virus infection, various health conditions and teratogen drugs; and environmental factors including air pollutants [4,5,6]. However, the etiology of most cases is still unknown [4]. Therefore, it is imperative to better understand the effects of genetic and environmental factors on the development of CHD in order to prevent CHD. *Methylenetetrahydrofolate reductase* gene (*MTHFR*) polymorphisms have been associated with many congenital anomalies including neural tube defects, deformed organs and body structures, and CHD [7]. *MTHFR* polymorphisms affects folate metabolism in the one carbon metabolism (OCM) and methylation pathways, with MTHFR enzymes involved in the homocysteine metabolism to prevent the accumulation of homocysteine [7,8]. Homocysteine inhibits the conversion of retinal to retinoic acid during the developing stage of fetal heart tetralogy, thus causing congenital defects [9]. Two of the most common polymorphisms involved with *MTHFR* are C677T (rs 1801133) and A1298C (rs 1801131). The *MTHFR* 677 TT homozygous and CT heterozygous polymorphisms result in 65% to 70% and 25% to 35% loss of the MTHFR enzyme function, respectively [10,11], whereas *MTHFR* 1298CC homozygous and AC heterozygous polymorphisms lead to a 20% and 10% loss of enzyme function, respectively, with an increased homocysteine level [11]. Individuals with *MTHFR* 677TT polymorphism have presented the greatest hyperhonocysteinemia and lower plasma folate levels as compared to those with other *MTHFR* polymorphisms, thus supplementation of folate has been suggested to individuals with *MTHFR* 677 TT genotype [12].

Previous meta-analyses presented that *MTHFR* C677T [13,14,15,16] and A1298C polymorphisms [13] were associated with the risk of CHD. However, other studies showed contradictive findings on these polymorphisms for children with CHD [17,18,19,20,21]. Furthermore, several meta-analysis studies presented an association between maternal *MTHFR* polymorphism and the risk of CHD for their children [13,14,18,22]; however, some studies presented inconsistent findings [15,20,21]. Only one previous meta-analysis study reported the association between paternal *MTHFR* polymorphisms and CHD, presenting *MTHFR* TT homozygous polymorphism as a risk genotype for CHD [16]. Therefore, while there have been an increasing number of studies to examine the association between *MTHFR* polymorphisms and the risk of CHD, there are still no conclusive results. Hence, a more comprehensive meta-analysis is necessary.

Previous studies demonstrated that air pollution deterred health outcomes for both generations of parents and children, affecting fetal development [6,23,24]. Air pollution was associated with impaired fetal development involving CHD, and increased infant mortality through methylation and inflammatory toxic ischemic pathways [25,26]. Additionally, air pollutants may impair the differentiation of neural crest cells [27], with free radicals generating superoxides, hydrogen peroxide, and hydroxyl radicals that impinge cell development [28]. Individuals with gene mutations in the methylation pathways have difficulty processing environmental toxicants [26,29,30]. The result is a progressive impairment in the conversion of key enzymes in the regulatory pathways, producing free radicals such as peroxynitrite and superoxides, and inflammatory substances [31]. During the last 15 years, many epidemiological studies investigated the association between air pollution and various forms of CHD [6,24,26,32,33]. The assessment of exposure presented great challenges [26], and the association between air pollution and CHD warrants further investigation.

In summary, previous meta-analyses published on *MTHFR* polymorphisms and CHD risk presented inconsistent results [13,14,15,16,17,18,19,20,21,22]. Additionally, no previous meta-analysis has been conducted to examine gene-environment interactions, specifically, the association of air pollution with *MTHFR* polymorphisms and the risks of CHD. Hence, we conducted meta-analysis and meta-prediction to examine the impact of air pollution on the *MTHFR* polymorphisms and the risk of CHD.

## 2. Materials and Methods

### 2.1. Search Strategy

Following the guidelines of meta-analysis of observational studies in epidemiology (MOOSE) [34] and preferred reporting items for systematic reviews and meta-analyses (PRISMA) [35], we conducted an online database search for relevant articles published up to 2017 in PubMed and Embase. We combined the following key words “congenital heart disease”, “heart defects, congenital”, “rs 1801133”, “rs 1801131”, “*MTHFR*”, and “*methylenetetrahydrofolate reductase*” and limited the results to human studies. Reference lists of previous meta-analysis were reviewed to identify additional articles. We entered the resulting articles into a database organized by key words. The entries were checked between two raters to ensure the accuracy of the data entries.

### 2.2. Inclusion and Exclusion Criteria-Study Identification

The inclusion criteria were the studies that: (1) examined the association between *MTHFR* C677T or A1298C polymorphisms and CHD; (2) were conducted by case-control and/or cross-sectional designs for children, mothers, or fathers; (3) and were written in English or in Chinese but provided an abstract in English and tables that clearly listed the genotype allele frequencies for both case and control groups. All seven non-English articles were written in Chinese language. And, the authors are proficient in Chinese. Therefore, there was no obstacle to extract the content of these articles. The exclusion criteria were the studies that: (1) Included the subjects with other congenital abnormality such as Down syndrome; (2) and did not offer *MTHFR* genotype allele counts or no appropriate genotype allele counts.

**Figure 1:**
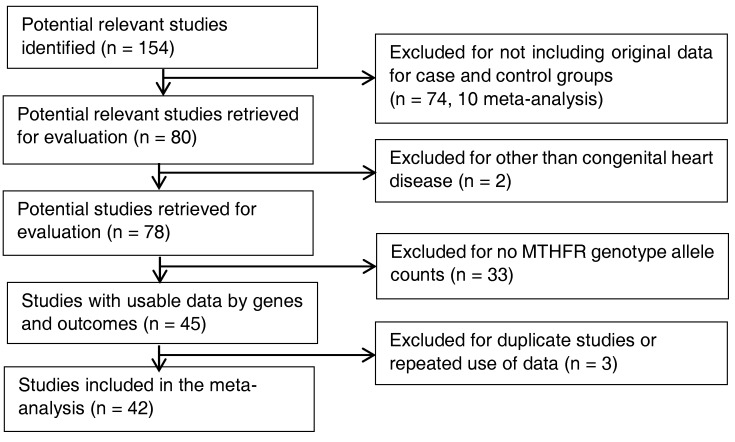
Progression on the selection of studies for the meta-analysis.

Based on the inclusion criteria using the same sets of key words and the mesh terms as listed under Section 2.1 above, we identified 154 studies involving *MTHFR* gene polymorphisms and CHD for screening (96 studies from PubMed, 45 studies from Embase databases, and 13 papers by cross references methods). A total of 74 articles were excluded for not being the original studies, with 10 being meta-analyses [13,14,15,16,17,18,19,20,21,22]. We further excluded two articles that presented other congenital defects such as Down syndrome, and 33 articles without *MTHFR* genotype allele counts per groups. Additionally, we carefully examined potential duplicate use of data from related publications using the same data and three articles were excluded. Finally, we included 42 articles for pooled analysis (Figure 1). All 42 articles contained *MTHFR* C677T genotype data, and 15 articles included *MTHFR* A1298C genotype data (Supplementary Table S1).

### 2.3. Quality Assessment

We examined inter-rater reliability by checking the rating results between the two raters. The deviations between raters were discussed to reach a consensus for consistency of data entry. We evaluated each study for quality using the indicators that were organized and examined by a previous study in the field [31]. The Newcastle-Ottawa Scale (NOS) was endorsed by Cochrane Collaboration to assess the quality of observation studies in the 2011 handbook, and it is easy to use with a limited exploration of quality [36,37]. However, the Cochrane Collaboration also acknowledged that researchers may add assessment of the quality that were not included in the NOS [38]. The quality assessment tool used in this meta-analysis was developed based on several quality assessment tools and covered all items of NOS, with six items specific to genotyping analysis that were used in this field of study [23,31,38,39,40]. Total quality score for this study ranged from 0–28 which included three sub-scores to obtain the total score: (1) External validity, with nine items on demographic data; (2) internal validity, with 12 items on research methods and procedures; and (3) seven items on data and results reporting. The quality score of ≥50% on the total possible score indicated that the findings were trustworthy [36].

### 2.4. Data Synthesis and Analysis

We used Excel spreadsheets (Microsoft Corp, Redmond, WA, USA) and StatsDirect, version 2.4.7 (Cheshire, UK) to pool the results of data analyses. We checked the Hardy-Weinberg Equilibrium (HWE) analysis, which was developed to assess the distribution equilibrium of the evolutionary mechanisms in population genetics [31,41,42]. Departure from the HWE with a *p*-value < 0.05 could potentially be associated with factors such as population migration or stratification and disease association (Supplementary Table S1). Because the total quality score of several studies scored <50% of the total possible score and some studies had significant HWE, we performed sensitivity analyses with subgroups which included and excluded these studies. The results of these subgroup analyses, however, did not yield significant differences for pooled analyses. Therefore, we included all 58 study groups in the analysis, consistent with the approaches used in recent meta-analyses in the field [31].

We calculated both odds ratios (ORs) and risk ratios (RRs) to perform pooled analysis and compared the differences between the two ratios. The results of ORs and RRs were similar, and the RRs were more conservative with less Type-1 errors. Additionally, RR includes all three genotypes for the ratios versus OR, which is only a pair-wise ratio, thus, RR has been used as the standardized ratio per consensus panel for gene-environment interaction analysis [23,43,44,45,46]. Therefore, we adopted RRs in this study; this method has also been used in recent meta-analysis studies [23,31,43]. We calculated pooled RRs for *MTHFR* polymorphism subtypes between cases and controls, and found 95% confidence intervals (CIs) for the associations of *MTHFR* polymorphism genotypes with CHD. The RR of 1 represents “no effect,” <1 indicates a protective effect (favoring the case, CHD group), and >1 indicates increased risks for CHD. We defined significant findings as those with *p*-values < 0.05. We performed the Q test and *I^2^* index to test the heterogeneity between studies. Random effects instead of fixed effects models were used for the risk estimates when the heterogeneity tests were significant with *p*-value < 0.05 in Q test or values of *I^2^* ≥ 50% [36]. The funnel plot, Begg’s and Egger’s test were used to investigate the publication bias. An asymmetric funnel plot and *p*-value < 0.05 in Begg’s and Egger’s test suggested a possible publication bias. No significant publication bias was found on the meta-analysis of the *MTHFR* polymorphism test (T = 0–0.181, bias = −0.547–0.333, all *p* > 0.05). We used the funnel plot of *MTHFR* TT plus CT as an example to show the symmetric distribution of the data (Supplementary Figure S1).

Because different racial-ethnic groups presented different polymorphism patterns, we performed subgroup analysis per racial-ethnic group. The categories of these racial-ethnic groups were presented in the included studies with subgroups of Caucasian, East Asian, South Asian, Mixed, Middle Eastern, Hispanic, and African. The data further revealed heterogeneity with regional differences on the percentage of polymorphisms and risks of CHD, thus, we used geographic information system (GIS) maps in JMP Pro (SAS Institute, Cary, NC, USA) to visualize the distributions of polymorphisms and risks on the global maps. Analyses were performed by both racial-ethnic group and geographical location because migration is common today and thus one can no longer equate a country or region to only one race or ethnic group. By doing so, a comprehensive picture that takes both genetic and environmental factors into account can be revealed. These GIS maps were drawn by country distribution and were helpful for visually identifying geographic patterns. In addition, we applied recursive partition trees in the JMP Pro program to examine how an independent variable (e.g., air pollution) can make a decisive split of the data by partitioning the groups (such as air pollution levels) into subgroups with reference to the dependent variable (the percentage of polymorphisms and CHD risks). The recursive partition tree does this by exhaustively searching all possible groupings [47]. The goodness of the partition can be judged by using Akaike’s information criterion correction (AICc). A smaller AICc suggests a better model for fitness [48,49]. Both GIS maps and recursive partition trees are common machine-learning-based big-data analytical techniques for handling multidimensional and/or large-scale datasets. Different from conventional hypothesis testing, these analytics do not start with a pre-determined hypothesis. Rather, data-driven pattern recognition plays the central role.

For triangulation purpose, we also employed a conventional multiple comparison procedure (Tukey test) to examine whether partition trees and Tukey tests concurred with each other. Furthermore, we used nonlinear curve fit to examine the associations between air pollution and the outcome variables. The aim of meta-predictive analysis was to generate more precise predictions while integrating data from diverse sources. The main purpose for using both conventional statistical and machine learning (e.g., recursive partition trees) methods is to verify the results by cross-validation with AICc [50]. We entered the air-quality data for various countries, using the guidelines from the World Health Organization on air quality measures and the death rates from air pollution (AP death) (Level 1: ≤50 deaths per million, Level 2: 51–100 deaths per million, Level 3: 101–250 deaths per million, Level 4: 251–400 deaths per million, and Level 5: ≥401 deaths per million) [51,52]. We further verified these levels with current scales on air pollution data [53,54,55,56] and used the most complete scaled air pollution data for the analyses. Only one study from Puerto Rico presented as Level 1, thus, we combined Level 1 and Level 2 together for grouping analysis. No study in this study was conducted in country presented Level 5 air pollution. Additionally, the risks of respiratory and circulatory system diseases increase with particulate matter (PM) [57,58]. However, current air pollution indexes including PM are not available for past times for all countries included in this study. Thus, we chose an outcome-based index (AP death) that is available across all included studies.

## 3. Results

### 3.1. Characteristics of Original Studies

For the *MTHFR* C677T genotype, 23 articles included data of children; eight articles of mothers; seven articles for each of children and mothers, yielding seven additional study groups; three articles for each of mothers and fathers but not children yielding three additional study groups; and one article for each of children, mothers, and fathers yielding two additional study groups [59]. Additionally, one study [60] included data for five racial ethnic groups in children, yielding four additional study groups. In summary, there were 42 articles containing 58 study groups (12,347 cases and 18,106 controls). These 58 study groups included 35 studies of children (9751 cases and 15,050 controls), 19 studies on mothers of children with CHD (2038 cases and 2560 controls), and four studies on fathers of children with CHD (558 cases and 496 controls) (Supplementary Table S1).

For *MTHFR* A1298C genotypes, eight articles included data of children; two articles of mothers; four articles for each of children and mothers yielding four additional study groups; and one article for each of children, mothers, and fathers, yielding two additional study groups. In summary, there were 15 articles containing 21 study groups (2754 cases and 3419 controls). These 21 study groups included 13 studies of children (1835 cases and 2003 controls), seven studies of mothers (691 cases and 1165 controls), and one study of fathers (228 cases and 251 controls) (Supplementary Table S1).

Study populations were drawn from countries across the globe, including Europe, North America, South America, East Asia, South Asia, Middle East Asia and Africa. We checked the racial-ethnic compositions of each study to ensure that we properly accounted for data from distinct versus mixed racial-ethnic groups per studies. The most investigated racial-ethnic populations in those studies were Asian (21 studies, including 20 East Asian and 1 Southeast Asian), followed by Caucasian (14 studies), then African (8 studies), mixed race (7 studies), Hispanics (5 studies), and Middle-East Asian (3 studies) (Supplementary Table S1).

### 3.2. Association between MTHFR *C677T* and Risk of CHD

We summarized significant associations between *MTHFR* C677T polymorphisms and CHD risks (Table 1). For all included studies, both generations, *MTHFR* 677TT and TT plus CT polymorphisms together were risk genotypes for CHD (RR = 1.30 and 1.07; 95% CI = 1.17–1.44 and 1.04–1.11) (Supplementary Table S2); and for subgroups of children (RR = 1.30 and 1.09; 1.14–1.48 and 1.04–1.14) (Table 2), parents (RR = 1.24 and 1.06; 1.09–1.41 and 1.01–1.011) (Supplementary Table S3a), and mothers (RR = 1.21 and 1.05; 1.04–1.39 and 1.00–1.11) (Supplementary Table S3b), while *MTHFR* 677TT homozygous polymorphism was the risk genotype for fathers (RR = 1.39, 1.02–1.91) (Supplementary Table S3c). Contrarily, *MTHFR* 677CC common allele type played a protective role against CHD for both generations (RR = 0.91, 0.87–0.96) (Supplementary Table S2); children (RR = 0.90, 0.85–0.96) (Table 2); parents (RR = 0.92, 0.87–0.98) (Supplementary Table S3a); and mothers (RR = 0.93, 0.87–1.00) (Supplementary Table S3b).

#### 3.2.1. Subgroup Analyses by Ethnic Groups for *MTHFR* C677T

For all studies combined in both generations of children and parents, subgroup analysis by ethnic group presented a much higher percentage of *MTHFR* 677TT in the case group than the control group in East Asian (24.1% versus 17.6%) and Hispanic (19.2% versus 15.6%) than other ethnic groups. *MTHFR* 677TT was the risk genotype for CHD in Caucasian and East Asian; and a potential risk genotype for African (Table 1, Supplementary Table S2). For the children’s subgroup, *MTHFR* 677TT was a risk type for CHD in East Asian and African. *MTHFR* 677 TT plus CT together were the risk types for CHD in Caucasian and East Asian (Table 2). For parental subgroup, *MTHFR* 677 TT was a risk type for CHD in East Asian and Hispanic (Supplementary Table S3a, see details for maternal subgroup in Supplementary Table S3b and paternal subgroup in Supplementary Table S3c).

#### 3.2.2. Subgroup Analyses by CHD Types for *MTHFR* C677T

For different types of CHD developed during the embryonic process [61,62], we further performed subgroup analyses for both generations. For children, the percentage of *MTHFR* 677TT in the CHD group was higher in cyanotic CHD (18.9%) than mixed (14.0%) and acyanotic CHDs (6.2%), and a risk type for mixed CHD (RR = 1.25) and cyanotic CHD (RR = 1.41, Supplementary Table S4a). For both parental and maternal subgroups, *MTHFR* 677TT was a risk type for both mixed CHD and cyanotic CHD (Supplementary Table S4b; Supplementary Table S4c). And, for the paternal subgroup, all studies were mixed CHD; *MTHFR* 677TT was a risk type in Caucasian fathers.

#### 3.2.3. Subgroup Analyses by Countries for *MTHFR* C677T

**Figure 2:**
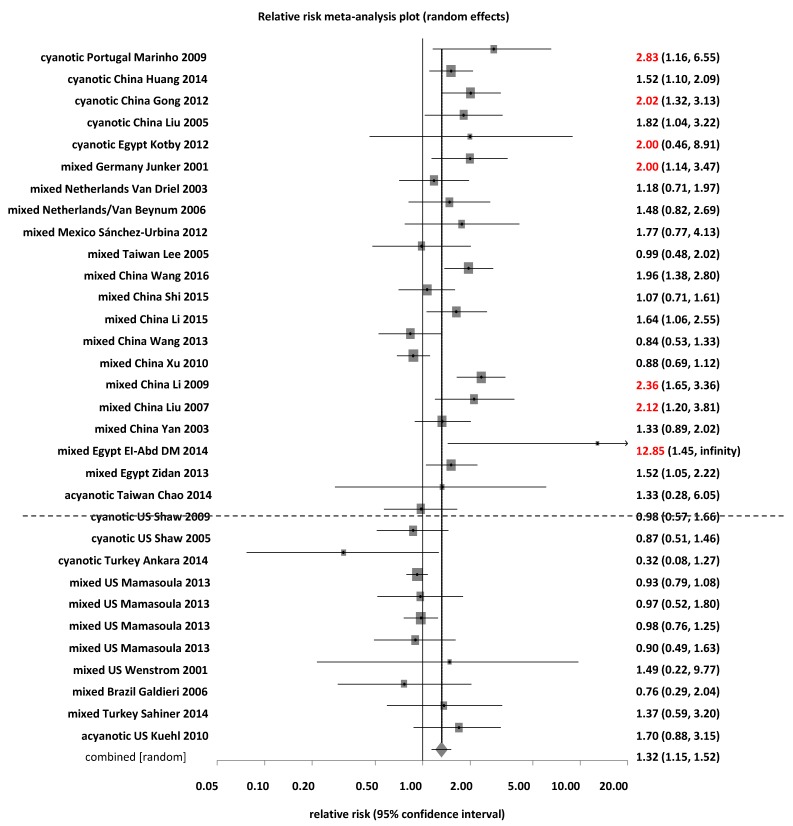
Forest plot of risks of congenital heart diseases (CHD) by *MTHFR* 677 TT polymorphisms per countries for children. Note. Cyanotic: cyanotic CHDs; mixed: cyanotic and acyanotic CHDs mixed; acyanotic: acyanotic CHDs; studies above dotted line: risk > 1; studies under dotted line: risk < 1; red words: risk > 2.

The distribution of *MTHFR* C677T polymorphisms per country for control and CHD groups varied across countries (Supplementary Figure S2). We used GIS maps to visualize geographical distributions of polymorphisms and CHD risks for regional patterns. We used the yellow-red color spectrum to show the percentage of *MTHFR* 677TT, and the green to red color spectrum to present the risk for CHD with red indicating risk and green indicating a protective effect (Supplementary Figure S3). Using the percentage of *MTHFR* 677TT for the children’ group as an example, the top three countries were Egypt (22.7%), China (19.5%) and Brazil (15.8%) in the control group (Supplementary Figure S2 left panel, Supplementary Figure S3a); and Egypt (38.2%), China (27.1%), and Mexico (20.0%) in the CHD group (Supplementary Figure S2 right panel, Supplementary Figure S3b). *MTHFR* 677TT was a risk genotype of CHD for children in rank order: Portugal (RR = 2.83), Germany (RR = 2.00), Mexico (RR = 1.77), Egypt (RR = 1.69), China (RR = 1.33), Netherlands (RR = 1.39), US and Turkey (RR = 0.95), and Brazil (RR = 0.76) (Figure 2, Supplementary Figure S3c).

Additionally, we pooled countries per RR > 1, <1, or varied with *MTHFR* 677TT as a risk genotype. For all groups combined, countries with RR > 1 included Russia, Germany, Italy, Netherlands, Portugal, Puerto Rico, Mexico, Brazil, Taiwan, China, and Egypt. Conversely, countries with TT as a protective genotype (RR < 1) included US and Turkey (Supplementary Table S2). For children, the countries with RR > 1 included Germany, Mexico, Netherlands, Portugal, China, Taiwan, and Egypt (Table 2, Figure 2). It is noteworthy that many countries presented with pooled RR > 2 for CHD risk from *MTHFR* 677TT, including Germany, Portugal, China and Egypt (7 studies). An RR > 2 inferred causality for health outcomes in biologic studies as a strong evidence by the consensus panels [44,45]. Countries with TT as a protective genotype (RR < 1) included Brazil, Turkey, and US (Table 2, Figure 2, Supplementary Figure S2c). For parents, the countries with RR > 1 included Netherlands (3 studies), Russia, Italy, US, Puerto Rico, Mexico (2 studies), Brazil, China (7 studies), and Egypt (4 studies) (Supplementary Table S3a). For maternal subgroup, the countries by risk per RR > 1 included Russia, Italy, US, Brazil, Puerto Rico, Mexico, China, and Egypt (Supplementary Table S3b, Supplementary Figure S4). Additionally, countries with studies presenting RR > 2 for CHD risk for mothers included Brazil, Puerto Rico, Mexico, China, and Egypt (6 studies). Countries with TT as a protective genotype (RR < 1) included Netherlands and Austria (Supplementary Table S3b, Supplementary Figure S4). For paternal subgroup, the RRs of all studies were >1, including countries of Austria, China, and Netherlands (Supplementary Table S3c, Supplementary Figure S4), and no studies from fathers’ data presented RR > 2.

### 3.3. Association between MTHFR A1298C and Risk of CHD

For a pooled analysis of *MTHFR* A1298C polymorphisms, we summarized significant findings in Table 3. For all included studies of both generations, *MTHFR* 1298CC and CC plus AC polymorphisms together were risk genotypes for CHD (RR = 1.44 and 1.16; 1.07–1.95 and 1.02–1.31) (Table 3, Supplementary Table S5a) and for children (RR = 1.56 and 1.20; 1.28–1.91 and 1.01–1.43) (Table 3, Supplementary Table S5b).

#### 3.3.1. Subgroup Analyses by Ethnic Groups and CHD Types for *MTHFR* A1298C

For both generations, subgroup analysis by ethnic subgroups presented much higher percentages of *MTHFR* 1298CC homozygous polymorphism in the case group than in the control group in South Asian (38.5% versus 22.0%), Middle Eastern (18.0% versus 9.9%), and African (54.5% versus 22.7%) than other ethnic groups. *MTHFR* 1298CC was a risk genotype for CHD in East Asian, Middle Eastern, and African (Table 3, Supplementary Table S5a). For children, the percentages of *MTHFR* 1298CC were much higher in the case group than in the control group in South Asian (38.5% versus 22.0%), Middle Eastern (18.0% versus 9.9%), and African (55.5% versus 23.6%) than other ethnic groups. *MTHFR* 1298CC was a risk type for CHD in Middle Eastern (Table 3, Supplementary Table S5b). For the parental subgroup, the percentage of *MTHFR* 1298CC was much higher in the case group than in the control group in African (53.6% versus 21.8%) than other ethnic groups. *MTHFR* 1298CC was a protective genotype for CHD in Caucasian while *MTHFR* 1298AA was a protective genotype for CHD in African (Table 3, Supplementary Table S5c). For the maternal subgroup, *MTHFR* 1298 CC plus AC were risk types for CHD in African (Table 3, Supplementary Table S5d).

#### 3.3.2. Subgroup Analysis by Countries for *MTHFR* A1298C

The distribution of *MTHFR* A1298C polymorphisms per countries for control and CHD case group varied across countries. Using the percentage of *MTHFR* 1298CC polymorphism for the children’ group as an example, the top three countries were Egypt (23.6%), India (22.0%), and Netherlands (10.0%) in the control group; and Egypt (55.58%), India (38.5%), and Turkey (18.0%) in the case group (Supplementary Figure S5). For all groups combined, countries with *MTHFR* 1298 CC RR > 1 included Italy, Taiwan, China, India, Turkey, and Egypt (Supplementary Table S5a, Supplementary Figure S6). Countries with CC as a protective genotype (RR < 1) included Russia, Netherlands, and Brazil for all groups combined (Supplementary Table S5a and Supplementary Figure S6). For children, countries with RR > 1 included Netherlands, Taiwan, India, Turkey, and Egypt (Supplementary Table S5b and Supplementary Figure S6). It is worthy to point out that countries with studies of RR > 2 for CHD risk from *MTHFR* 1298CC for children included Taiwan, Turkey, and Egypt (Three studies, Supplementary Figure S5). Contrarily, *MTHFR* 1298CC was a protective genotype (RR < 1) in Brazilian children (Supplementary Table S5b). For the parental subgroup, countries with RR > 1, *MTHFR* 1298CC as a risk genotype, included Italy, Brazil, China, and Egypt (Supplementary Table S5a). Contrarily, *MTHFR* 1298CC was a protective genotype (RR < 1) in Russia and Netherlands (Supplementary Table S5c). For maternal subgroup, countries with RR > 1 included Italy, Brazil, China, and Egypt (Supplementary Table S5d and Supplementary Figure S5). Additionally, countries with studies presenting RR > 2 for CHD risk included Brazil, China, and Egypt (three studies, Supplementary Figure S6). Countries with CC homozygous as a protective genotype (RR < 1) included Russia and Netherlands for mothers (Supplementary Table S5d).

### 3.4. Meta-Prediction

**Figure 3:**
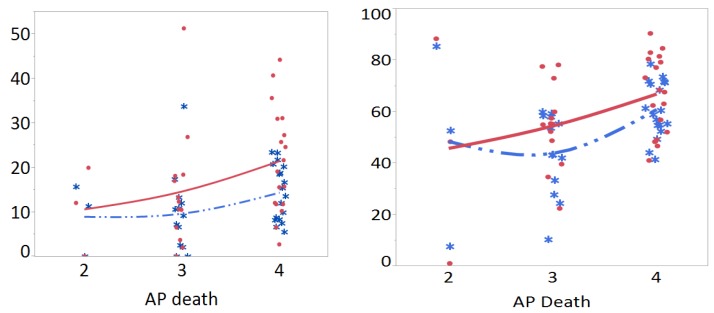
Nonlinear fit on percentage of *MTHFR* 677 TT (**left**) and *MTHFR* 677 TT + CT (**right**) polymorphisms for control (blue line) and congenital heart disease (red line) groups in association with death from air pollution for children. Note. AP death: Death rates from air pollution, Levels per million: 2: <100 (India, Mexico, and Brazil), 3: 100–250 (Germany, US, Iran, Egypt), 4: >250 (Portugal, Netherlands, China, Taiwan, Turkey).

We focused meta-prediction analysis on association of air pollution with *MTHFR* polymorphism and CHD risk in children and parents separately to see the generational effects. Literally “meta” means “above” and meta-prediction means generating a final predictive model on top of many predictive models. This “divide and conquer” strategy is also known as Split/Analyze/Meta-Analyze (SAM) approach [63]. We first used nonlinear fit to visualize the associations between air pollution presented with AP death levels and the percentage of *MTHFR* 677 TT polymorphisms (Figure 3 left panel) per CHD case (red line) and control (blue line) groups in children. The percentage of *MTHFR* 677TT in the case group was constantly higher than that in the control group. Additionally, the percentage of *MTHFR* 677TT increased with the increased AP death levels in the case group; whereas the percentage of *MTHFR* 677TT was flat between level 2 and level 3 and increased at level 4 AP death levels in the control group. For *MTHFR* 677 TT plus CT polymorphisms together (Figure 3 right panel), the percentage of polymorphisms gradually increased with increasing AP death levels in the case group; whereas, a slight decline was noted from level 2 to level 3 on AP death in the control group.

We then analyzed the association of air pollution with *MTHFR* 677 polymorphisms and CHD risk (Table 4) using both partition trees (split groups) and the Tukey’s tests. For children, the partition tree split the data for the percentage of *MTHFR* 677TT, CT, and TT plus CT polymorphisms into two groups by AP death levels 2–4. The results of Tukey’s tests were consistent with the findings of partition trees on the differences of *MTHFR* 677 polymorphisms between AP death levels 2–4 (AICcs ranged from 237 to 300) (Table 4 and Supplementary Figure S7). Specifically, the Tukey’s tests presented significant increases of *MTHFR* 677CT and TT plus CT polymorphisms with increased air pollution levels between AP death Levels 3 and 4 (difference = 12.36% and 17.14% respectively, both *p* < 0.05) for the control group (Table 4). On the risk of CHD from the *MTHFR* 677 polymorphism, there was a trend of increased CHD risk with a higher level of air pollution; the partition tree split the risk of TT polymorphism into two groups by AP death levels, with Level 4 presenting higher risk (RR = 1.50) than the Levels 2 and 3 combined (RR = 1.30), with smaller AICc (ranged from 56 to 62) for the partition tree tests (smaller the better). There were no significant findings on the parental subgroup for the association of air pollution with *MTHFR* polymorphisms and CHD, and on the association of air pollution with *MTHFR* A1298C polymorphism and CHD risk for any groups.

## 4. Discussion

We presented a comprehensive meta-analysis for transgenerational effects of *MTHFR* polymorphisms of C677T and A1298C in association with the development of CHD, involving children and parents. With meta-predictive analysis, we demonstrated that air pollution was associated with the increased percentage of *MTHFR* 677 TT homozygous and *MTHFR* 677 TT plus CT polymorphisms together, and the trends of increased risks of CHD with TT polymorphisms in children. *MTHFR* 677 TT homozygous polymorphism was a risk genotype for CHD across all study groups in both generations combined, and subgroups of children, mothers, and fathers. Additionally, *MTHFR* 677 TT plus CT polymorphisms together were risk genotypes for all groups combined in both generations and subgroups of children and mothers, whereas, *MTHFR* 677 CC common allele type played a protective role against CHD. These polymorphisms carried significance in ethnic subgroups of Caucasian, East Asian, and African. Furthermore, we found both generations’ *MTHFR* 1298 CC and CC plus AC polymorphisms together were risk types for CHD, and also significant for subgroups of children: East Asian, Middle Eastern, and African. These results demonstrated the significance of *MTHFR* polymorphisms and associated enzyme functions in the development of CHD for future prevention efforts.

Air pollution was associated with CHD [6,24,64]. Several potential mechanisms altering a correct heart morphogenesis included the methylation pathway and toxic inflammatory ischemic pathway with placental hemorrhage, fetal death, and embryo resorption [65]. *MTHFR* polymorphisms play an essential role in the methylation pathway decreasing folate levels and causing hyper-homocysteinemia. Additionally, air pollution may affect normal differentiation of neural crest cells which are important for heart development [27] and are sensitive to toxic insults with apoptosis [28]. We demonstrated that air pollution was associated with increased *MTHFR* 677 polymorphisms, with a more profound impact in countries with higher levels of air pollution, especially in East Asian countries, China particularly. These findings are consistent with prior studies associating increased air pollution levels with increased *MTHFR* gene polymorphisms in various cancers, Alzheimer’s disease, and hypertensive disorders during pregnancy (HDP) [23,31,38,39,40]. Additionally, increased air pollution levels were associated with *MTHFR* polymorphism-associated HDP risk [23]. Further studies are imperative to examine the association between air pollution and CHD.

For meta-prediction-associated meta-regression analyses, linear modeling, such as Pearson’s correlation and ordinary least squares regression, have been criticized as overly simplistic. One of the classic examples is presented by Anscombe [66], who claimed that it failed to detect curvilinear association at the inflection point. By the same token, it is a common misconception that as air pollution gets worse, more people die, but there is an inflection point in this association. When the underlying data structure is nonlinear, such as the association between death and air pollution, then nonlinear modeling is more appropriate [67,68,69]. In addition, in spite of the potential risk of ecological fallacy, in some cases, global data must be used because collecting individual data is impossible. Air pollution is a typical example that presents a challenge in measuring the assessment of pollution exposure [26]. Although techniques of monitoring how much pollution is absorbed by each individual has been under development by taking immediate surroundings, an individual’s biophysical characteristics, and an individual’s space-time activities into account [70]; these methods are not prevalent across countries. Thus, future studies must continue to overcome these methodological challenges in assessing the exposure effects on health outcomes.

Our results presented that both maternal *MTHFR* 677 *TT* and *MTHFR* 677 TT plus CT and paternal *MTHFR* 677 TT polymorphisms were significant risk genotypes for CHD, demonstrating a greater and more extensive impact of maternal than paternal polymorphisms on the CHD risk [59]. Maternal *MTHFR* 677 TT polymorphism was also a risk type for East Asian and Hispanic, while paternal *MTHFR* 677 TT polymorphism was a risk type for Caucasian. Our results demonstrated that *MTHFR* polymorphisms were associated with all subtypes of CHD for children [71], versus previous analyses which presented the associations with different types of CHD [5,72]. Additionally, our pooled analyses demonstrated the effects of *MTHFR* A1298C polymorphism on CHD risks, while previous meta-analyses demonstrated inconsistent results on such an association [13,15,17], as we included more studies than prior analyses.

It is noteworthy that many countries presented CHD risk with RR > 2 from *MTHFR* polymorphisms of both 677 and 1298, which inferred causality in biologic studies as strong evidence by the consensus panels [44,45]. These countries with high risk of RR > 2 included Germany, Portugal, China, and Egypt (seven studies) for children; and Brazil, Puerto Rico, Mexico, China, and Egypt (six studies) for mothers from *MTHFR* 677 polymorphisms (13/58, 22.4% of the included studies). Whereas, countries with greater risk of CHD from *MTHFR* 1298 polymorphisms included Taiwan, Turkey, and Egypt (three studies) for children; and Brazil, China, and Egypt (three studies) for mothers (6/21, 28.6% of included studies). The number of studies with high risk of disease risk (RR > 2) resulted from *MTHFR* polymorphisms, which are greater in this meta-analysis of CHD condition than in previous meta-analyses of pregnant women with hypertensive diseases (15/71 studies, 21.1% of included studies for *MTHFR* 677; 2/11 studies, 18.2% of the included studies for *MTHFR* 1298) [23] and Alzheimer’s diseases (8/43, 18.6% of the studies for *MTHFR* 677) [38]. The mechanisms of such heightened risk impact on CHD than other diseases from *MTHFR* polymorphisms are worthy of further investigations on the transgenerational effects for epigenetic methylation pathways. Future studies could be conducted to examine the effects of gene-environment interactions between gene polymorphisms and air pollution on the health outcomes across generations and lifespan. The results of heightened disease risks for children with CHD could bring attention to health policy for a clean air environment and interventions to mitigate the enzyme deficiency in the folate metabolism pathways for parents with *MTHFR* polymorphisms to prevent CHDs for future generations.

Several limitations should be considered when interpreting these findings. First, the air pollution measure presented great challenges in the assessment of exposure [26,70] and the availability of data across all countries over time. We used the most validated air pollution measure available for all countries examined in this study—the death from air pollution. However, future studies must continue to examine the measurement on air pollution and its effects on health outcomes. Second, the quality score of four studies in the current analysis was not optimal, <50% of the total possible score. However, we included these studies due to no significant differences being found on the parameters tested after conducting the sensitivity analysis. Third, the potential covariates of individual characteristics including parental health problems or health behaviors were not reported in the original studies for further testing; thus, these parental data could not be controlled or tested. Further studies can continue to test these parameters to contribute to this field of study. Additionally, future studies could focus on examining the interventions to supplement folate and essential nutrients in the methylation pathways to prevent CHDs for childbearing-age parents, especially for those presenting health conditions with compromised folate-metabolism. 

## 5. Conclusions

We applied a comprehensive meta-analysis and found that both *MTHFR* C677T and A1298C gene polymorphisms were risks for CHD, with transgenerational effects. Additionally, meta-prediction analysis revealed the percentage of *MTHFR* 677TT and TT plus CT polymorphisms together were increased in countries with higher levels of air pollution, with a trend of increased CHD risks with higher levels of air pollution for children. Our findings may have significant implications on advocating for a clean-air environment for public health policy and future intervention studies to mitigate the folate-related enzyme deficits in the folate-related metabolism pathways resulting from *MTHFR* polymorphisms which prevent CHDs for future generations.

## Figures and Tables

**Table 1:** Schema of significant findings across studies on *MTHFR* 677 genotypes and risk of congenital heart diseases (CHD).

*MTHFR* 677	All	Children	Parents	Mothers	Fathers
	58 studies (*n* Case/*n* Control) (12,347/18,106)	35 Studies (9751/15,050)	23 Studies (2596/3056)	19 Studies (2038/2560)	4 Studies (558/496)
Overall (58 Studies)	Risk Type: TT, TT + CT Protective: CC	Risk Type: TT, TT + CT Protective: CC	Risk Type: TT, TT + CT Protective: CC	Risk Type: TT, TT + CT Protective: CC	Risk Type: TT
Subgroups					
Caucasian (14 Studies)	14 Studies (5923/9998) Risk Type: TT, TT + CT	7 Studies (5096/8626) Risk Type: TT + CT	7 studies (827/1372) NS	5 studies (567/1091) NS	2 studies (260/281) Risk Type: TT
East Asian (20 Studies)	20 Studies (3139/3113) Risk Type: TT, TT + CT Protective: CC	13 Studies (2259/2345) Risk Type: TT, TT + CT Protective: CC	7 studies (880/768) Risk Type: TT	5 studies (582/553) Risk Type: TT	2 studies (298/215) NS
South Asian (1 Study)	1 Study (96/90) --	1 Study (96/90) --	--	--	--
Mixed (7 Studies)	7 Studies (1268/1634) NS	5 Studies (668/1252) NS	2 studies (600/382) NS	2 studies (600/382) NS	--
Middle Eastern (3 Studies)	3 Studies (334/313) NS	3 Studies (334/313) NS	--	--	--
Hispanic (5 Studies)	5 Studies (626/1326) NS	2 Studies (508/982) NS	3 studies (118/344) Risk Type: TT	3 studies (118/344) Risk Type: TT	--
African (8 Studies)	8 Studies (961/1632) Risk Type: TT, TT + CT Protective: CC	4 Studies (790/1442) Risk Type: TT	4 studies (171/190) NS	4 studies (171/190) NS	--

Note: Only one study for African mothers on acyanotic CHD; NS: No Statistical Significance; -- No data.

**Table 2:** Pooled analysis: *MTHFR* 677 genotypes and risks of congenital heart diseases for children (35 Studies).

Genotypes by Race or Ethnicity (Number of Studies)	Case (*N* = 9751) *n* (%)		Control (*N* = 15,050) *n* (%)		Test of Heterogeneity			Statistical Model	Test of Association		
					Q	*p*	I^2^ %		Risk Ratio (95% Cl)		*p*
TT (35)	1402	(14.4)	1791	(12.9)	85.2	<0.0001	62.4	Random	1.30	(1.14–1.48)	0.0001
Caucasian (7)	605	(11.9)	1005	(11.7)	16.3	0.0123	63.1	Random	1.23	(0.98–1.53)	0.069
East Asian (13)	566	(25.1)	429	(18.3)	39.4	<0.0001	69.6	Random	1.46	(1.17–1.82)	0.0008
South Asian (1)	0	(0.0)	0	(0.0)	-	-	-	-	0	-	-
Mixed (5)	61	(9.1)	117	(9.3)	0.39	0.9831	0	Fixed	0.91	(0.67–1.23)	0.54
Middle Eastern (3)	16	(4.8)	15	(4.8)	2.7	0.0989	63.6	Fixed	0.89	(0.43–1.81)	0.74
Hispanic (2)	88	(17.3)	167	(17.0)	1.7	0.1923	41.2	Fixed	1.03	(0.81–1.30)	0.84
African (4)	66	(8.4)	58	(4.0)	3.7	0.2997	18.2	Fixed	1.44	(1.05–1.98)	0.026
CT (35)	4180	(42.9)	6311	(41.9)	52.3	0.0023	35.0	Random	1.00	(0.96–1.05)	0.98
Caucasian (7)	2318	(45.5)	3837	(44.5)	10.2	0.1154	41.3	Fixed	1.03	(0.99–1.07)	0.16
East Asian (13)	1065	(47.1)	1108	(47.2)	18.8	0.0936	36.1	Fixed	0.99	(0.93–1.05)	0.70
South Asian (1)	1	(1.0)	7	(7.8)	-	-	-	-	0.13	-	-
Mixed (5)	258	(28.6)	474	(37.6)	10.5	0.0333	61.8	Random	1.04	(0.84–1.31)	0.70
Middle Eastern (3)	146	(43.7)	137	(43.8)	1.0	0.5939	0	Fixed	1.01	(0.85–1.21)	0.89
Hispanic (2)	213	(41.9)	421	(42.9)	0.0	0.8641	0	Fixed	0.94	(0.83–1.06)	0.32
African (4)	179	(22.7)	327	(22.7)	4.8	0.1884	37.3	Fixed	0.96	(0.82–1.13)	0.65
CC (35)	4169	(42.8)	6948	(46.2)	124.0	<0.0001	72.6	Random	0.90	(0.85–0.96)	0.0014
Caucasian (7)	2173	(42.6)	3784	(43.9)	17.8	0.0066	66.4	Random	0.93	(0.84–1.03)	0.16
East Asian (13)	628	(27.8)	808	(34.5)	50.6	<0.0001	76.3	Random	0.75	(0.62–0.91)	0.0028
South Asian (1)	95	(99.0)	83	(92.2)	-	-	-	-	1.07	-	-
Mixed (5)	349	(52.5)	661	(52.8)	5.9	0.2079	32	Fixed	1.02	(0.93–1.11)	0.61
Middle Eastern (3)	172	(51.5)	161	(51.4)	1.9	0.3910	0	Fixed	1.00	(0.86–1.16)	0.996
Hispanic (2)	207	(40.7)	394	(40.1)	0.4	0.5497	0	Fixed	1.06	(0.93–1.20)	0.39
African (4)	545	(69.0)	1057	(73.3)	19.2	0.0002	84.4	Random	0.64	(0.38–1.06)	0.083
TT + CT (35)	5582	(57.2)	8102	(53.8)	115.6	<0.0001	70.6	Random	1.09	(1.04–1.14)	0.0008
Caucasian (7)	2923	(57.4)	4842	(56.1)	25.9	0.0002	76.9	Random	1.09	(1.00–1.19)	0.0427
East Asian (13)	1631	(72.2)	1537	(65.5)	42.6	<0.0001	71.9	Random	1.13	(1.04–1.21)	0.0022
South Asian (1)	1	(1.0)	7	(7.8)	-	-	-	-	0.13	-	-
Mixed (5)	319	(47.8)	591	(47.2)	10.7	0.0295	62.8	Random	1.01	(0.84–1.22)	0.92
Middle Eastern (3)	162	(48.5)	152	(48.6)	1.8	0.3990	0	Fixed	1.00	(0.85–1.17)	0.996
Hispanic (2)	301	(59.3)	588	(59.9)	1.1	0.3013	6.4	Fixed	0.96	(0.88–1.05)	0.395
African (4)	245	(31.0)	385	(26.7)	14.9	0.0019	79.9	Random	1.35	(0.93–1.94)	0.11
Subgroups											
TT risk > 1 (7 countries)	3001	(43.8)	3485	(32.0)							
TT (21)	704	(23.5)	543	(15.6)	46.3	0.0007	56.8	Random	1.51	(1.28–1.79)	<0.0001
CT (21)	1384	(46.1)	1601	(45.9)	25.7	0.1755	22.2	Fixed	0.99	(0.94–1.05)	0.8242
CC (21)	913	(30.4)	1341	(38.5)	66.6	<0.0001	70.0	Random	0.76	(0.66–0.87)	<0.0001
TT + CT (21)	2088	(69.6)	2144	(61.5)	58.3	<0.0001	65.7	Random	1.14	(1.07–1.21)	<0.0001
TT risk < 1 (3 countries)	3624	(53.0)	7182	(66.0)							
TT (11)	373	(10.3)	781	(10.9)	6.4	0.7776	0	Fixed	0.95	(0.84–1.07)	0.37
CT (11)	1397	(38.5)	2833	(39.4)	17.8	0.0580	43.9	Fixed	0.98	(0.929–1.03)	0.34
CC (11)	1854	(51.2)	3568	(49.7)	15.2	0.1239	34.3	Fixed	1.03	(0.99–1.07)	0.13
TT + CT (11)	1770	(48.8)	3614	(50.3)	30.3	0.0008	67	Random	1.00	(0.92–1.10)	0.97
TT risk varied (2 countries)	219	(3.2)	215	(2.0)							
TT (2)	0	(0.0)	0	(0.0)	-	-	-	-	-	-	-
CT (2)	61	(27.9)	61	(28.4)	4.3	0.0384	76.7	Random	0.50	(0.06–4.08)	0.50
CC (2)	158	(72.1)	154	(71.6)	6.0	0.0139	83.5	Random	1.00	(0.74–1.33)	0.98
TT + CT (2)	61	(27.9)	61	(28.4)	4.3	0.0384	76.7	Random	0.50	(0.06–4.08)	0.51

Note. Q: Cochran’s Q; CI: confidence interval. TT risk > 1 (7 countries): China (11 studies), Egypt (3 studies), Germany, Mexico, Netherlands (2 studies), Portugal, and Taiwan (2 studies); TT risk < 1 (3 countries) Brazil, Turkey (2 studies), and US (8 studies); TT risk cannot be determined (2 countries): India and Iran; One study included sample from multiple countries in Europe.

**Table 3:** Schema of significant findings across studies on *MTHFR* 1298 genotypes and risk of congenital heart diseases (CHD).

*MTHFR* A1298C	All	Children	Parents	Mothers
	21 Studies (*n* Case/*n* Control) (2754/3419)	13 Studies (1835/2003)	8 Studies (919/1416)	7 Studies (691/1165)
Overall (21 Studies)	Risk Type: CC, CC + AC	Risk Type: CC, CC + AC	NS	NS
Subgroups				
Caucasian (5 Studies)	5 Studies (838/1315) Protective: AC	1 Study (229/251) --	4 Studies (609/1064) Protective: CC	3 Studies (381/813) NS
East Asian (7 Studies)	7 Studies (1290/1528) Risk Type: CC	6 Studies (1137/1312) NS	1 Study (153/216) --	1 Study (153/216) --
South Asian (1 Study)	1 Study (96/100) --	1 Study (96/100) --	0 --	0 --
Mixed (2 Studies)	2 Studies (104/64) NS	1 Study (57/38) --	1 Study (47/26) --	1 Study (47/26) --
Middle Eastern (2 Studies)	2 Studies (206/192) Risk Type: CC	2 Studies (206/192) Risk Type: CC	0 --	0 --
African (4 Studies)	4 Studies (220/220) Risk Type: CC, CC + AC Protective: AA	2 Studies (110/110) Risk Type: CC + AC Protective: AA	2 Studies (110/110) Risk Type: CC + AC Protective: AA	2 Studies (110/110) Risk Type: CC + AC Protective: AA

Note. Only one study for fathers; NS: No Statistical Significance; --: No data.

**Table 4:** Meta-prediction: Death from air pollution on *MTHFR* 677 genotypes for controls and congenital heart diseases (CHD) cases, and CHD risks for children (35 studies).

		Partition Tree						Tukey Test			
Variable	AICc	AP Death	Count	Mean	SD	Levels Compared	D	SED	Lower CI	Upper CI	*p*
TT % ct	237.40	2, 3	16	9.58	8.58	4/2	5.42	4.69	−6.13	16.97	0.49
		4	18	14.45	6.19	4/3	4.75	2.74	−1.99	11.49	0.21
						3/2	0.67	4.82	−11.2	12.53	0.99
TT % CHD	271.23	2, 3	16	13.95	12.54	4/2	10.86	7.69	−8.06	29.78	0.35
		4	18	21.57	11.86	4/3	6.87	4.49	−4.17	17.91	0.29
						3/2	3.99	7.89	−15.44	23.42	0.87
CT % ct	271.28	3, 2	16	35.16	16.10	4/3	12.36	4.47	1.36	23.37	0.03
		4	18	46.51	7.14	4/2	6.91	7.66	−11.94	25.76	0.64
						2/3	5.45	7.87	−13.91	24.82	0.77
CT % CHD	271.16	2, 3	16	39.00	15.60	4/2	10.26	7.67	−8.61	29.13	0.39
		4	18	45.42	8.00	4/3	5.54	4.47	−5.47	16.56	0.44
						3/2	4.72	7.87	−14.66	24.10	0.82
TT + CT % ct	289.28	2, 3	16	44.72	20.19	4/3	17.14	5.85	2.73	31.55	0.02
		4	18	60.96	10.74	4/2	12.33	10.03	−12.36	37.02	0.45
						2/3	4.81	10.30	−20.55	30.16	0.89
TT + CT %	300.01	2, 3	16	52.94	21.86	4/2	21.11	11.69	−7.66	49.88	0.18
CHD		4	18	66.99	15.16	4/3	12.42	6.82	−4.37	29.21	0.18
						3/2	8.69	12.01	−20.86	38.23	0.75
RR TT	56.12	2, 3	13	1.30	0.46	4/2	0.24	0.42	−0.80	1.28	0.84
		4	18	1.50	0.61	4/3	0.20	0.22	−0.33	0.73	0.63
						3/2	0.04	0.43	−1.03	1.11	0.996
RR CT	62.47	2, 3	16	1.23	0.81	3/2	0.68	0.35	−0.18	1.54	0.14
		4	18	0.98	0.15	3/4	0.37	0.20	−0.11	0.86	0.16
						4/2	0.31	0.34	−0.53	1.14	0.64
RR TT + CT	57.95	2, 3	16	1.29	0.76	3/2	0.74	0.32	−0.05	1.52	0.07
		4	18	1.10	0.15	4/2	0.40	0.31	−0.36	1.17	0.41
						3/4	0.33	0.18	−0.11	0.78	0.18

Note. RR: risk ratio; AP death: Death rates from air pollution, levels per million: 2: <100 (India, Mexico, and Brazil), 3: 100–250 (Germany, US, Iran, Egypt), 4: >250 (Portugal, Netherlands, China, Taiwan, Turkey); ct: control group; D: Difference; SED: SE Difference; AP death was split by partitioning the original group into pairs of subgroups with reference to the dependent variable (e.g., the percentage of polymorphism and CHD risks) by recursive partition trees in the JMP 13 program (SAS Institute).

## Supplementary Materials

The following are available online at http://www.mdpi.com/1660-4601/15/8/1660/s1, Supplementary Table S1. Characteristics of studies in the world for MTHFR 677 and 1298 loci distributions (42 papers). Supplementary Table S2. Pooled analysis: MTHFR 677 genotype and risk of congenital heart disease for all study groups (58 studies). Supplementary Table S3a. Pooled analysis: MTHFR 677 genotypes and risks of congenital heart diseases for parents (23 Studies). Supplementary Table S3b. Pooled analysis: MTHFR 677 genotype and risk of congenital heart disease for mothers (19 studies). Supplementary Table S3c. Pooled analysis: MTHFR 677 genotype and risk of congenital heart disease for fathers (4 studies). Supplementary Table S4a. Pooled analysis: MTHFR C677T polymorphism and risks of congenital heart disease (CHD) per CHD types for children (35 study groups). Supplementary Table S4b. Pooled analysis: MTHFR C677T polymorphism and risks of congenital heart disease (CHD) per CHD types for parents (23 studies). Supplementary Table S4c. Pooled analysis: MTHFR C677T polymorphism and risks of congenital heart disease (CHD) per CHD types for mothers (19 studies). Supplementary Table S5a. Pooled analysis: MTHFR 1298 genotype and risk of congenital heart diseases for all study groups (21 studies). Supplementary Table S5b. Pooled analysis: MTHFR 1298 genotype and risk of congenital heart disease for children (13 studies). Supplementary Table S5c. Pooled analysis: MTHFR 1298 genotype and risk of congenital heart diseases for parents (8 studies). Supplementary Table S5d. Supplementary Figure S1. The Funnel plot of MTHFR 677 TT + CT. Supplementary Figure S2. The percent of MTHFR 677 TT and CT polymorphism per control (left) and congenital heart diseases case (right) groups. Supplementary Figure S3. Geographic information maps for the percent of MTHFR 677 TT polymorphism (a) per control group (ct), (b) per congenital heart disease (CHD) group (ca), and (c) CHD risk for children. Supplementary Figure S4. Forest plot of risks of congenital heart disease (CHD) by MTHFR 677 TT polymorphisms by types of CHD for mothers and fathers. Supplementary Figure S5. The percent of MTHFR 1298 CC and AC polymorphism per control (left side) and congenital heart disease case (right side) groups. Supplementary Figure S6. Forest plot of risks of congenital heart disease (CHD) by MTHFR 1298 CC polymorphisms per countries for children. Supplementary Figure S7. Recursive partition tree: the percent of MTHFR TT polymorphism by death from air pollution (AP death) for children with congenital heart disease.
