# Supplementary material for: Meta-Prediction of MTHFR Gene Polymorphism and Air Pollution on the Risks of Congenital Heart Defects Worldwide: A Transgenerational Analysis
Source: Int J Environ Res Public Health. 2018 Aug 5;15(8):1660. doi: 10.3390/ijerph15081660 (PMC6121605; doi:10.3390/ijerph15081660)
Supplement: Supplementary file 1 [file ijerph-15-01660-s001.pdf]

Supplementary Table 1. Characteristics of studies in the world for MTHFR 677 and 1298 loci distributions (42 papers).

First Author (Reference number)[1]	Year	Ethnicity - Country	Subject	*MTHFR* C677T									*MTHFR A1298C*							Quality score[5]
				Case, disease type (DT), n (%)				Control, Source of control (SOC), n (%)					Case n (%)			Control n (%)				
				DT[2]	CC	CT	TT	SOC[3]	CC	CT	TT	HWE[4]	AA	AC	CC	AA	AC	CC	HWE	
**Europe**																				
Mamasoula [14]	2013	Caucasian - 5 European countries[6]	Children	2	1244 (42.8)	1338 (46.0)	325 (11.2)	1	1885 (45.2)	1816 (43.6)	467 (11.2)	Yes	NA							15 (3, 5, 7)
Weiner [15]	2012	Caucasian - Russia	Mothers	2	18 (40.0)	21 (46.7)	6 (13.3)	3	173 (49.7)	149 (42.8)	26 (7.5)	Yes	33 (68.8)	13 (27.1)	2 (4.2)	168 (46.4)	152 (42.0)	42 (11.6)	Yes	21 (7, 9, 5)
Wintner [16]	2007	Caucasian - Austria	Mother	2	17 (54.8)	11 (35.5)	3 (9.7)	3	10 (32.3)	17 (54.8)	4 (12.9)	Yes	NA							22 (6, 9, 7)
			Fathers	2	17 (54.8)	11 (35.5)	3 (9.7)	5	14 (46.7)	14 (46.7)	2 (6.7)	Yes								22 (6, 9, 7)
Junker [17]	2001	Caucasian - Germany	Children	2	51 (44.7)	42 (36.8)	21 (18.4)	1	129 (56.6)	78 (34.2)	21 (9.2)	Yes	NA							19 (7, 8, 4)
Storti [18]	2003	Caucasian - Italy	Mothers	1	27 (26.2)	53 (51.5)	23 (22.3)	3	52 26.0)	108 (54.0)	40 (20.0)	Yes	49 (47.6)	46 (44.7)	8 (7.8)	101 (50.5)	86 (43.0)	13 (6.5)	Yes	20 (6, 8, 6)
van Driel [19]	2008	Caucasian - Netherlands	Children	2	99 (43.2)	103 (45.0)	27 (11.8)	1	119 (47.4)	107 (42.6)	25 (10.0)	Yes	112 (48.9)	90 (39.3)	27 (11.8)	97 (38.6)	129 (51.4)	25 (10.0)	Yes	26 (7, 12, 7)
			Mothers	2	91 (39.6)	117 (50.9)	22 (9.6)	3	111 (44.2)	104 (41.4)	36 (14.3)	Yes	104 (45.2)	102 (44.3)	24 (10.4)	116 (46.2)	104 (41.4)	31 (12.4)	Yes	26 (7, 12, 7)
			Fathers	2	105 (45.9)	94 (41.0)	30 (13.1)	5	129 (51.4)	103 (41.0)	19 (7.6)	Yes	111 (48.7)	98 (43.0)	19 (8.3)	91 (36.3)	123 (49.0)	37 (14.7)	Yes	26 (7, 12, 7)
van Beynum [20]	2006	Caucasian - Netherlands	Children	2	79 (47.9)	66 (40.0)	20 (12.1)	1	98 (47.3)	104 (47.3)	18 (8.2)	No	NA							21 (7, 8, 7)
			Mothers	2	72 (45.6)	68 (43.0)	18 (11.4)	3	131 (50.2)	107 (41.0)	23 (8.8)	Yes	NA							21 (7, 8, 7)
Marinho [21]	2009	Caucasian - Portugal	Children	1	12 (31.6)	20 (52.6)	6 (15.8)	1	113 (45.0)	124 (49.4)	14 (5.6)	No	NA							8 (3, 2, 3)
North America																				
Mamasoula [14]	2013	Caucasian - USA	Children	2	676 (42.6)	716 (45.1)	196 (12.3)	1	1306 (40.7)	1474 (45.9)	428 (13.3)	Yes	NA							20 (5, 9, 6)
Mamasoula [14]	2013	Hispanic -USA	Children	2	200 (44.6)	172 (38.4)	76 (17.0)	1	385 (41.8)	375 (40.8)	160 (17.4)	No	NA							20 (5, 9, 6)
Mamasoula [14]]	2013	Mixed -USA	Children	2	131 (60.4)	72 (33.2)	14 (6.5)	1	258 (57.8)	156 (35.0)	32 (7.2)	Yes	NA							20 (5, 9, 6)
Mamasoula [14]	2013	African-USA	Children	2	508 (77.7)	132 (20.2)	14 (2.1)	1	992 (75.5)	293 (22.3)	29 (2.2)	Yes	NA							20 (5, 9, 6)

First Author (Reference number)[1]	Year	Ethnicity - Country	Subject	*MTHFR* C677T									*MTHFR A1298C*							Quality score[5]
				Case, disease type (DT), n (%)				Control, Source of control (SOC), n (%)					Case n (%)			Control n (%)				
				DT[2]	CC	CT	TT	SOC[3]	CC	CT	TT	HWE[4]	AA	AC	CC	AA	AC	CC	HWE	
Kuehl [22]	2010	Caucasian - USA	Children	3	12 (21.6)	33 (60.0)	10 (18.2)	2	134 (44.7)	134 (44.7)	32 (10.7)	Yes	NA							21 (4, 10, 7)
Hobbs [23]	2010	Mixed -USA	Mothers	2	285 (51.5)	203 (36.7)	65 (11.8)	3	191 (53.7)	128 (36.0)	37 (10.4)	No	NA							22 (6, 9, 7)
Shaw [24]	2009	Mixed -USA	Children	1	102 (47.7)	89 (41.6)	23 (10.7)	1	101 (46.3)	93 (42.7)	24 (11.0)	Yes	NA							20 (6, 8, 6)
Shaw [25]	2005	Mixed -USA	Children	1	69 (45.1)	68 (44.4)	16 (10.5)	1	180 (41.5)	202 (46.5)	52 (12.0)	Yes	NA							21 (7, 8, 6)
Wenstrom [26]	2001	Mixed -USA	Children	2	17 (65.4)	8 (30.8)	1 (3.8)	1	104 (89.7)	9 (7.8)	3 (2.6)	No	NA							18 (6, 7, 5)
García-Fragoso [27]	2010	Hispanic - Puerto Rico	Mothers	2	10 (37.0)	11 (40.7)	6 (22.2)	7	84 (38.2)	115 (52.3)	21 (9.5)	No	NA							15 (5, 5, 5)
Balderrábano-Saucedo [28]	2013	Hispanic - Mexico	Mothers	1	7 (22.6)	12 (38.7)	12 (38.7)	3	24 (38.7)	31 (50.0)	7 (11.3)	Yes	NA							19 (6, 8, 5)
Sánchez-Urbina [29]	2012	Hispanic - Mexico	Children	2	7 (11.7)	41 (68.3)	12 (20.0)	1	9 (14.5)	46 (74.2)	7 (11.3)	No	NA							17 (3, 8, 6)
			Mothers	2	8 (13.3)	38 (63.3)	14 (23.3)	3	13 (21.0)	37 (59.7)	12 (19.4)	Yes								17 (3, 8, 6)
**South America**																				
Galdieri [30]	2006	Mixed - Brazil	Children	2	30 (51.7)	21 (36.2)	7 (12.1)	1	18 (47.4)	14 (36.8)	6 (15.8)	Yes	35 (61.4)	21 (36.8)	1 (1.8)	19 (50.0)	16 (42.1)	3 (7.9)	Yes	16 (6, 5, 5)
			Mothers	2	27 (57.4)	15 (31.9)	5 (10.6)	1	10 (38.5)	15 (57.7)	1 (3.8)	Yes	26 (55.3)	17 (36.2)	4 (8.5)	15 (57.7)	10 (38.5)	1 (3.8)	Yes	16 (7, 4, 5)
**East Asia**																				
Chao [31]	2014	East Asian - Taiwan	Children	3	10 (58.8)	5 (29.4)	2 (11.8)	1	19 (55.9)	12 (35.3)	3 (8.8)	Yes	13 (76.5)	2 (11.8)	2 (11.8)	15 (44.1)	19 (55.9)	0 (0)	No	17 (6, 6, 5)
Lee [32]	2005	East Asian - Taiwan	Children	2	110 (51.6)	89 (41.8)	14 (6.6)	1	114 (58.5)	68 (34.9)	13 (6.7)	Yes	NA							17 (3, 8, 6)
Wang [33]	2016	East Asian - China	Children	2	14 (9.5)	73 (49.7)	60 (40.8)	1	49 (29.2)	84 (50.0)	35 (20.8)	Yes	NA							23 (6, 11, 6)
Shi [34]	2015	East Asian - China	Children	2	37 (26.8)	71 (51.4)	30 (21.7)	1	80 (38.6)	85 (41.1)	42 (20.3)	No	74 (53.6)	57 (41.3)	7 (5.1)	163 (78.7)	36 (17.4)	8 (3.9)	No	20 (2, 11, 7
			Mothers	2	55 (35.9)	68 (44.4)	30 (19.6)	3	70 (32.4)	101 (46.8)	45 (20.8)	Yes	95 (62.1)	39 (25.5)	19 (12.4)	157 (72.7)	53 (24.5)	6 (2.8)	Yes	21 (3, 11, 7)
Li [35]	2015	East Asian - China	Children	2	31 (20.7)	78 (52.0)	41 (27.3)	1	59 (39.3)	66 (44.0)	25 (16.7)	Yes	114 (76.0)	36 (24.0)	0 (0)	131 (87.3)	19 (12.7)	0 (0)	Yes	22 (8, 8, 6)
Jiang [36]	2015	East Asian - China	Mothers	2	38 (38.0)	46 (46.0)	16 (16.0)	2	41 (41.0)	48 (48.0)	11	Yes	NA							22 (6, 10, 6)

First Author (Reference number)[1]	Year	Ethnicity - Country	Subject	*MTHFR* C677T									*MTHFR A1298C*							Quality score[5]
				Case, disease type (DT), n (%)				Control, Source of control (SOC), n (%)					Case n (%)			Control n (%)				
				DT[2]	CC	CT	TT	SOC[3]	CC	CT	TT	HWE[4]	AA	AC	CC	AA	AC	CC	HWE	
Huang [37]	2014	East Asian - China	Children	1	63 (37.5)	45 (26.8)	60 (35.7)	2	84 (41.2)	72 (35.3)	48 (23.5)	No	111 (65.3)	56 (32.9)	3 (1.8)	146 (70.9)	54 (26.2)	6 (2.9)	Yes	21 (6, 9, 6)
Wang [38]	2013	East Asian - China	Children	2	59 (36.9)	76 (47.5)	25 (15.6)	1	53 (28.2)	100 (53.2)	35 (18.6)	Yes	115 (71.9)	40 (25.0)	5 (3.1)	133 (70.7)	47 (25.0)	8 (4.3)	Yes	21 (4, 11, 6)
Gong [39]	2012	East Asian - China	Children	1	45 (18.4)	123 (50.4)	76 (31.1)	2	43 (31.6)	72 (52.9)	21 (15.4)	Yes	NA							23 (6, 10, 7)
Xu [40]	2010	East Asian - China	Children	2	162 (32.3)	244 (48.6)	96 (19.1)	2	151 (28.7)	261 (49.5)	115 (21.8)	Yes	316 (62.9)	168 (33.5)	18 (3.6)	326 (61.9)	185 (35.1)	16 (3.0)	Yes	22 (7, 9, 6)
Li [41]	2009	East Asian - China	Children	2	16 (15.4)	42 (40.4)	46 (44.2)	2	55 (26.4)	114 (54.8)	39 (18.8)	Yes	NA							20 (6, 8, 6)
Liu [42]	2007	East Asian - China	Children	2	30 (22.7)	68 (51.5)	34 (25.8)	1	46 (43.0)	48 (44.9)	13 (12.1)	Yes	NA							17 (6, 5, 6)
Zhong [43]	2006	East Asian - China	Mothers	2	67 (58.3)	33 (28.7)	15 (13.0)	4	76 (66.1)	34 (29.6)	5 (4.3)	Yes	NA							15 (5, 7, 3)
			Fathers	2	67 (58.3)	41 (35.7)	7 (6.1)	6	73 (63.5)	34 (29.6)	8 (7.0)	Yes	NA							15 (5, 7, 3)
Liu [44]	2005	East Asian - China	Children	1	19 (19.6)	54 (55.7)	24 (24.7)	1	33 (28.0)	69 (58.5)	16 (13.6)	No	NA							12 (4, 4, 4)
Yan [45]	2003	East Asian - China	Children	2	32 (17.1)	97 (51.9)	58 (31.0)	1	22 (21.4)	57 (55.3)	24 (23.3)	Yes	NA							12 (4, 3, 5)
Yan [46]	2003	East Asian - China	Mothers	2	32 (17.1)	94 (50.3)	61 (32.6)	4	20 (19.6)	57 (55.9)	25 (24.5)	Yes	NA							11 (4, 2, 5)
			Fathers	2	26 (14.2)	104 (56.8)	53 (29.0)	6	21 (21.0)	57 (57.0)	22 (22.0)	Yes	NA							11 (4, 2, 5)
Liu [47]	2002	East Asian - China	Mothers	2	5 (18.5)	14 (51.9)	8 (29.6)	3	2 (10.0)	15 (75.0)	3 (15.0)	No	NA							14 (6, 5, 3)
**South Asia**																				
Koshy [48]	2015	South Asian - India	Children	1	95 (99.0)	1 (1.0)	0 (0)	1	83 (92.2)	7 (7.8)	0 (0)	Yes	27 (28.1)	32 (33.3)	37 (38.5)	58 (58.0)	20 (20.0)	22 (22.0)	No	16 (6, 6, 4)
Middle-East Asia																				
Pishva [49]	2013	M-Eastern- Iran	Children	3	63 (51.2)	60 (48.8)	0 (0)	1	71 (56.8)	54 (43.2)	0 (0)	No	-	-	-	-	-	-	-	20 (5, 10, 5)
Sahiner [50]	2014	M-Eastern- Turkey	Children	2	69 (50.7)	53 (39.0)	14 (10.3)	1	47 (50.5)	39 (41.9)	7 (7.5)	Yes	45 (32.8)	68 (49.6)	24 (17.5)	31 (33.3)	54 (58.1)	8 (8.6)	No	15 (5, 4, 6)
Kocakap [51]	2014	M-Eastern - Turkey	Children	1	40 (53.3)	33 (44.0)	2 (2.7)	2	43 (45.3)	44 (46.3)	8 (8.4)	Yes	20 (29.0)	36 (52.2)	13 (18.8)	51 (51.5)	37 (37.4)	11 (11.1)	Yes	14 (5, 4, 5)
**Africa**																				

First Author (Reference number)[1]	Year	Ethnicity - Country	Subject	*MTHFR* C677T									*MTHFR A1298C*							Quality score[5]
				Case, disease type (DT), n (%)				Control, Source of control (SOC), n (%)					Case n (%)			Control n (%)				
				DT[2]	CC	CT	TT	SOC[3]	CC	CT	TT	HWE[4]	AA	AC	CC	AA	AC	CC	HWE	
Ei-Abd [52]	2014	African -Egypt	Children	2	7 (26.9)	12 (46.2)	7 (26.9)	1	13 (72.2)	5 (27.8)	0 (0)	Yes	NA							17 (4, 7, 6)
		African -Egypt	Mothers	2	10 (38.5)	15 (57.7)	1 (0)	3	9 (50.0)	9 (50.0)	0 (0)	Yes	NA							17 (4, 7, 6)
Elsayed [53]	2014	African -Egypt	Mothers	3	19 (54.3)	16 (45.7)	0 (0)	3	30 (49.2)	24 (39.3)	7 (11.5)	Yes	NA							21 (6, 10, 5)
Zidan [54]	2013	African -Egypt	Children	2	18 (22.5)	21 (26.3)	41 (51.3)	1	32 (40.0)	21 (26.3)	27 (33.8)	No	16 (20.0)	27 (33.8)	37 (46.3)	30 (37.5)	26 (32.5)	24 (30.0)	No	16 (5, 5, 6)
			Mothers	2	21 (26.3)	30 (37.5)	29 (36.3)	3	31 (38.8)	25 (31.3)	24 (30.0)	No	13 (16.3)	32 (40.0)	35 (43.8)	33 (41.3)	25 (31.3)	22 (27.5)	No	16 (5, 5, 6)
Kotby [55]	2012	African -Egypt	Children	1	12 (40.0)	14 (46.7)	4 (13.3)	1	20 (66.7)	8 (26.7)	2 (6.7)	Yes	2 (6.7)	4 (13.3)	24 (80.0)	12 (40.0)	16 (53.3)	2 (6.7)	Yes	19 (6, 8, 5)
			Mothers	1	12 (40.0)	16 (53.3)	2 (6.7)	3	20 (64.5)	10 (32.3)	1 (3.2)	Yes	2 (6.7)	4 (13.3)	24 (80.0)	14 (46.7)	14 (46.7)	2 (6.7)	Yes	18 (5, 8, 5)

[1] Reference numbers refer to the Reference List that follows this table.

[2] DT: Disease type, 1: cyanotic congenital heart disease (CHD) (n = 12), 2: mixed CHD (n = 42), 3: acyanotic CHD (n = 4).

[3] SOC: Sources of control, 1: healthy children (n = 30); 2: children without CHD (n = 7), 3: mother of healthy children (n = 14), 4: mother of children without CHD (n = 2), 5: father of healthy children (n = 2), 6: father of children without CHD (n = 2), 7: healthy volunteer (n = 1).

[4] HWE: Hardy Weinberg Equilibrium, updated from the original report based on our calculations using the formula available at http://www.koonec.com/k-blog/2010/06/20/hardy-weinberg-equilibrium-calculator.

[5] Quality score ranges: 0 - 28 (External validity, 0 - 9; Internal validity, 0 - 12; Report quality, 0 - 7).

[6] 5 European Countries: Australia, Belgium, Germany, Netherlands, and United Kingdom.

Reference List of Studies included in the Meta-analysis

Ten meta-analysis papers

1. Xuan, C.; Li, H.; Zhao, J.-X.; Wang, H.-W.; Wang, Y.; Ning, C.P.; Liu, Z.; Zhang, B.B.; He, G.W.; Lun, L.M. Association between mthfr polymorphisms and congenital heart disease: A meta-analysis based on 9,329 cases and 15,076 controls. *Sci Rep* **2014**, *4*, 7311. DOI: 10.1038/srep07311. [PubMed] [CrossRef]
2. Zhang, T.; Wu, Q. MTHFR C677T and A1298C polymorphisms are not related to ventricular or atrial septal defect: A meta-analysis of 1272 cases and 1386 controls. *Int J Clin Exp Med* **2016**, *9*, 10673-10683.
3. Chen, K.; Chen, L.; Li, W.; Fang, Y.; Huang, G. Maternal MTHFR C677T polymorphism and congenital heart defect risk in the Chinese Han population: A meta-analysis. *Genet Mol Res* **2013**, 12, 6212-6219. DOI: 10.4238/2013. [PubMed] [CrossRef]
4. Wang, W.; Wang, Y.; Gong, F.; Zhu, W.; Fu, S. MTHFR C677T polymorphism and risk of congenital heart defects: Evidence from 29 case-control and TDT studies. *PLoS One* **2013**, 8, e58041. DOI: 10.1371/journal.pone.0058041. [PubMed] [CrossRef]
5. Yin, M.; Dong, L.; Zheng, J.; Zhang, H.; Liu, J.; Xu, Z. Meta analysis of the association between mthfr c677t polymorphism and the risk of congenital heart defects. *Ann Hum Genet* **2012**, 76, 9-16. DOI: 10.1111/j.1469-1809.2011.00687.x. [PubMed] [CrossRef]
6. Nie, Y.; Gu, H.; Gong, J.; Wang, J.; Gong, D.; Cong, X.; Chen, X.; Hu, S. Methylenetetrahydrofolate reductase C677T polymorphism and congenital heart disease: A meta-analysis. Clin Chem Lab Med **2011**, 49, 2101-2108. DOI: 10.1515/CCLM.2011.673. [PubMed] [CrossRef]
7. Li, Z.; Jun, Y.; Zhong-Bao, R.; Jie, L.; Jian-Ming, L. Association between MTHFR C677T polymorphism and congenital heart disease. ***Herz*** **2015**, 40, 160-167. DOI: 10.1007/s00059-014-4144-8. [PubMed] [CrossRef]
8. Wang, W.; Hou, Z.; Wang, C.; Wei, C.; Li, Y.; Jiang, L. Association between 5, 10-methylenetetrahydrofolate reductase (MTHFR) polymorphisms and congenital heart disease: A meta-analysis. *Meta gene* **2013**, 1, 109-125. DOI: 10.1016/j.mgene.2013.09.009. [PubMed]
9. Van Beynum, I.; Den Heijer, M.; Blom, H.; Kapusta, L. The MTHFR 677C→ T polymorphism and the risk of congenital heart defects: A literature review and meta-analysis. *QJM* **2007**, 100, 743-753. DOI: 10.1093/qjmed/hcm094. [PubMed] [CrossRef]
10. Verkleij-Hagoort, A.; Bliek, J.; Sayed-Tabatabaei, F.; Ursem, N.; Steegers, E.; Steegers-Theunissen, R. Hyperhomocysteinemia and mthfr polymorphisms in association with orofacial clefts and congenital heart defects: A meta-analysis. DOI: 10.1002/ajmg.a.31684. *Am J Med Genet A* **2007**, 143, 952-960. [PubMed] [CrossRef]

Three papers with duplicate use of data on genotype allele counts

11. Obermann-Borst, S.A.; van Driel, L.M.; Helbing, W.A.; de Jonge, R.; Wildhagen, M.F.; Steegers, E.A.; Steegers-Theunissen, R.P. Congenital heart defects and biomarkers of methylation in childrenren: A case - control study. *Eur J Clin Invest* **2011**, 41, 143-150. DOI: 10.1111/j.1365-2362.2010.02388.x. [PubMed] [CrossRef]
12. Zhu, W.L.; Li, Y.; Yan, L.; Dao, J.; Li, S. Maternal and offspring mthfr gene C677T polymorphism as predictors of congenital atrial septal defect and patent ductus arteriosus. *Mol Hum Reprod* **2005**, 12, 51-54. DOI: 10.1093/molehr/gah252. [PubMed] [CrossRef]
13. Li, Y.; Cheng, J.; Zhu, W.; Dao, J.; Yan, L.; Li, M.; Li, S. Study of serum hcy and polymorphisms of hcy metabolic enzymes in 192 families affected by congenital heart disease. Beijing da xue xue bao. Yi xue ban= Journal of Peking University. *Health sci* **2005**, 37, 75-80. [PubMed]

Forty-two papers included in meta-analysis

One meta-analysis paper with individual data

14. Mamasoula, C.; Prentice, R.R.; Pierscionek, T.; Pangilinan, F.; Mills, J.L.; Druschel, C.; Pass, K.; Russell, M.W.; Hall, D.; Töpf, A. Association between c677t polymorphism of methylene tetrahydrofolate reductase and congenital heart disease: **meta-analysis** of 7697 cases and 13,125 controls.. *Circ Cardiovasc Genet* **2013**, *6*, 347-353. DOI: 10.1161/CIRCGENETICS.113.000191. [PubMed] [CrossRef]

Forty-one individual studies

15. Weiner, A.S.; Gordeeva, L.A.; Voronina, E.N.; Boyarskikh, U.A.; Shabaldin, A.V.; Filipenko, M.L. Polymorphisms in folate-metabolizing genes and risk of having an offspring with congenital anomalies in the west siberian region of Russia: A case–control study. *Prenat Diagn* **2012**, *32*, 1041-1048. DOI: 10.1002/pd.3952. [PubMed] [CrossRef]
16. Wintner, S.; Hafner, E.; Stonek, F.; Stuempflen, I.; Metzenbauer, M.; Philipp, K. Association of congenital cardiac defects and the c677t methylenetetrahydrofolate reductase polymorphism. *Prenat Diagn* **2007**, *27*, 704-708. DOI: 10.1002/pd.1761. [PubMed] [CrossRef]

17. Junker, R.; Kotthoff, S.; Vielhaber, H.; Halimeh, S.; Kosch, A.; Koch, H.G.; Kassenböhmer, R.; Heineking, B.; Nowak-Göttl, U. Infant methylenetetrahydrofolate reductase 677TT genotype is a risk factor for congenital heart disease. *Cardiovasc Res* **2001**, *51*, 251-254. [PubMed]
18. Storti, S.; Vittorini, S.; Iascone, M.R.; Sacchelli, M.; Collavoli, A.; Ripoli, A.; Cocchi, G.; Biagini, A.; Clerico, A. Association between 5, 10-methylenetetrahydrofolate reductase c677t and a1298c polymorphisms and conotruncal heart defects. *Clin Chem Lab Med* **2003**, *41*, 276-280. DOI: 10.1515/CCLM.2003.043. [PubMed] [CrossRef]
19. van Driel, L.M.; Verkleij-Hagoort, A.C.; de Jonge, R.; Uitterlinden, A.G.; Steegers, E.A.; van Duijn, C.M.; Steegers-Theunissen, R.P. Two MTHFR polymorphisms, maternal B-vitamin intake, and CHDs. *Birth Defects Res A Clin Mol Teratol* **2008**, *82*, 474-481. DOI: 10.1002/bdra.20463. [PubMed] [CrossRef]
20. van Beynum, I.M.; Kapusta, L.; den Heijer, M.; Vermeulen, S.H.; Kouwenberg, M.; Daniëls, O.; Blom, H.J. Maternal MTHFR 677C> T is a risk factor for congenital heart defects: Effect modification by periconceptional folate supplementation. *Eur Heart J* **2006**, *27*, 981-987. DOI: 10.1093/eurheartj/ehi815. [PubMed] [CrossRef]
21. Marinho, C.; Alho, I.; Guerra, A.; Rego, C.; Areias, J.; Bicho, M. The methylenetetrahydrofolate reductase gene variant (C677T) as a susceptibility gene for Tetralogy of Fallot. *Rev Port Cardiol* **2009**, *28*, 809-812. [PubMed]
22. Kuehl, K.; Loffredo, C.; Lammer, E.J.; Iovannisci, D.M.; Shaw, G.M. Association of congenital cardiovascular malformations with 33 single nucleotide polymorphisms of selected cardiovascular disease–related genes. *Birth Defects Res A Clin Mol Teratol* **2010**, *88*, 101-110. DOI: 10.1002/bdra.20630. [PubMed] [CrossRef]
23. Hobbs, C.A.; Cleves, M.A.; Karim, M.A.; Zhao, W.; MacLeod, S.L. Maternal folate-related gene environment interactions and congenital heart defects. *Obstet Gynecol* **2010**, *116*, 316-322. DOI: 10.1097/AOG.0b013e3181e80979. [PubMed] [CrossRef]
24. Shaw, G.M.; Lu, W.; Zhu, H.; Yang, W.; Briggs, F.B.; Carmichael, S.L.; Barcellos, L.F.; Lammer, E.J.; Finnell, R.H. 118 snps of folate-related genes and risks of spina bifida and conotruncal heart defects. *BMC Med Genet* **2009**, *10*, 49-60. DOI: 10.1186/1471-2350-10-49. [PubMed] [CrossRef]
25. Shaw, G.M.; Iovannisci, D.M.; Yang, W.; Finnell, R.H.; Carmichael, S.L.; Cheng, S.; Lammer, E.J. Risks of human conotruncal heart defects associated with 32 single nucleotide polymorphisms of selected cardiovascular disease-related genes. *Am J Med Genet A* **2005**, *138*, 21-26. DOI: 10.1002/ajmg.a.30924. [PubMed]
26. Wenstrom, K.D.; Johanning, G.L.; Johnston, K.E.; DuBard, M. Association of the C677T methylenetetrahydrofolate reductase mutation and elevated homocysteine levels with congenital cardiac malformations. *Am J Obstet Gynecol* **2001**, *184*, 806-817. DOI: 10.1067/mob.2001.113845. [PubMed] [CrossRef]
27. García-Fragoso, L.; García-García, I.; Leavitt, G.; Renta, J.; Ayala, M.A.; Cadilla, C.L. MTHFR polymorphisms in puerto rican childrenren with isolated congenital heart disease and their mothers. *Int J Genet Mol Biol* **2010**, *2*, 43-47. [PubMed]
28. Balderrábano-Saucedo, N.A.; Sánchez-Urbina, R.; Sierra-Ramírez, J.A.; García-Hernández, N.; Sánchez-Boiso, A.; Klunder-Klunder, M.; Arenas-Aranda, D.; Bravo-Hernández, G.; Noriega-Zapata, P.; Vizcaíno-Alarcón, A. Polymorphism 677C→ T MTHFR gene in Mexican mothers of childrenren with complex congenital heart disease. *Pediatr Cardiol* **2013**, *34*, 46-51. DOI: 10.1007/s00246-012-0380-y. [PubMed] [CrossRef]
29. Sánchez-Urbina, R.; Galaviz-Hernández, C.; Sierra-Ramírez, J.A.; Rangel-Villalobos, H.; Torres-Saldúa, R.; Alva-Espinoza, C.; de Lourdes Ramírez-Dueñas, M.; García-Cavazos, R.; Arámbula-Meraz, E. Methylenetetrahydrofolate reductase gene 677CT polymorphism and isolated congenital heart disease in a mexican population. *Revista Española de Cardiología (English Edition)* **2012**, *65*, 158-163. DOI: 10.1016/j.recesp.2011.09.022. [PubMed] [CrossRef]
30. Galdieri, L.C.; Arrieta, S.R.; Silva, C.M.; Pedra, C.A.; D'Almeida, V. Homocysteine concentrations and molecular analysis in patients with congenital heart defects. *Arch Med Res* **2007**, *38*, 212-218. DOI: 10.1016/j.arcmed.2006.09.012. [PubMed] [CrossRef]
31. Chao, C.S.; Wei, J.; Huang, H.W.; Yang, S.C. Correlation between methyltetrahydrofolate reductase (MTHFR) polymorphisms and isolated patent ductus arteriosus in taiwan. *Heart, Lung and Circulation* **2014**, *23*, 655-660. DOI: 10.1016/j.hlc.2014.01.010. [PubMed]
32. Lee, C.N.; Su, Y.N.; Cheng, W.F.; Lin, M.T.; Wang, J.K.; Wu, M.H.; Hsieh, F.J. Association of the c677t methylenetetrahydrofolate reductase mutation with congenital heart diseases. *Acta Obstet Gynecol Scand* **2005**, *84*, 1134-1140. DOI: 10.1111/j.0001-6349.2005.00611.x . [PubMed]
33. Wang, Y.; Zhang, H.; Yue, S.; Zhang, K.; Wang, H.; Dong, R.; Yang, X.; Liu, Y.; Ma, Y. Evaluation of high resolution melting for MTHFR C677T genotyping in congenital heart disease. *PloS One* **2016**, *11*, e0151140. DOI: 10.1371/journal.pone.0151140. eCollection 2016. [PubMed]
34. Shi, H.; Yang, S.; Liu, Y.; Huang, P.; Lin, N.; Sun, X.; Yu, R.; Zhang, Y.; Qin, Y.; Wang, L. Study on environmental causes and SNPs of MTHFR, MS and CBS genes related to congenital heart disease. *PloS One* **2015**, *10*, e0128646. DOI: 10.1371/journal.pone.0128646. eCollection 2015. [PubMed]
35. Li, D.; Yu, K.; Ma, Y.; Liu, Y.; Ji, L. Correlationship between congenital heart disease and polymorphism of MTHFR gene. *Wei Sheng Yan Jiu* **2015**, *44*, 933-938. [PubMed]
36. Jiang, Y.; Mei, J.; Zhang, W.; Qian, X.; Zhang, S.; Liu, C.; Yang, H. Correlation between offspring congenital heart disease and mthfr 677 C/T polymorphism and general status of pregnant women. *Zhonghua liu xing bing xue za zhi= Zhonghua Liuxingbingxue Zazhi* **2015**, *36*, 1072-1076. [PubMed]

37. Huang, J.; Mei, J.; Jiang, L.; Jiang, Z.; Liu, H.; Ding, F. Mthfr rs1801133 C> T polymorphism is associated with an increased risk of tetralogy of Fallot. *Biomed Rep* **2014**, *2*, 172-176. DOI: 10.3892/br.2014.222. [PubMed]
38. Wang, B.; Liu, M.; Yan, W.; Mao, J.; Jiang, D.; Li, H.; Chen, Y. Association of snps in genes involved in folate metabolism with the risk of congenital heart disease. *J Matern Fetal Neonatal Med* **2013**, *26*, 1768-1777. DOI: 10.3109/14767058.2013.799648. [PubMed]
39. Gong, D.; Gu, H.; Zhang, Y.J.; Gong, J.; Nie, Y.; Wang, J.; Zhang, H.; Liu, R.; Hu, S.; Zhang, H. Methylenetetrahydrofolate reductase C677T and reduced folate carrier 80 G > A polymorphisms are associated with an increased risk of conotruncal heart defects. *Clin Chem Lab Med* **2012**, *50*, 1455-1461. DOI: 10.1515/cclm-2011-0759. [PubMed]
40. Xu, J.; Xu, X.; Xue, L.; Liu, X.; Gu, H.; Cao, H.; Qiu, W.; Hu, Z.; Shen, H.; Chen, Y. Mthfr c. 1793 G> A polymorphism is associated with congenital cardiac disease in a chinese population. *Cardiol Young* **2010**, *20*, 318-326. DOI: 10.1017/S1047951110000247. [PubMed]
41. Li, D.; Jing, X.; Wang, H.; Ye, W.; Fan, H. Study of correlationship between congenital heart disease and 5, 10-methylenetetra hydrofolate reductase gene's polymorphism or folacin intakes. *Zhonghua yu fang yi xue za zhi [Chinese journal of preventive medicine]* **2009**, *43*, 700-704. [PubMed]
42. Liu, Y.; Yin, X.; Wang, J.; Yu, L.; Liu, H.; Meng, F.; Liu, D. Relationship between genetic polymorphism of homocysteine metabolism enzymes and congenital heart disease. *Chinese Journal of Cardiovascular Review* **2007**, *5*, 210-213. [CrossRef]
43. Zhong, Q.; Qiu, X.; Zeng, X.; Lin, N. Association of congenital heart diseases with MTHFR gene and CBS gene. *Guangxi Medical Journal* **2006**, *8*, 1140-1142.
44. Liu, F.; Bai, P.; Chen, S.; Qiu, W.; Liu, X.; Zhang, Y. Association between 5, 10-methylenetetrahydrofolate reductase c677t polymorphisms and conotruncal heart defects in chinese childrenren. *Chin J Contemp Pediatr* **2005**, *7*, 99-102.
45. Ying, Y.; Li, Y. MTHFR C677T polymorphism and congenital heart disease. *Journal of Peking University (Health Sciences)* **2003**, *35*, 448-402.
46. Yan, L.; Li, S.; Zhao, H.; Zhao, R.; Dao, J.; Zhu, W.; Li, Y. Effect of 5, 10-methylenetetrahydro-folate reductase genotypes in parents on the risk of congenital heart disease in offspring. *Chin J Control Prev* **2003**, *7*, 94-97.
47. Liu, H.; Li, S., L.; Hongmao, M. Y. Maternal homocysteine folic acid, MTHFR gene polymorphism and congenital heart defects in offspring. *Chin J Perinat Med* **2002**, *5*, 102-105. [CrossRef]
48. Koshy, T.; Venkatesan, V.; Perumal, V.; Hegde, S.; Paul, S.F.D. The A1298C methylenetetrahydrofolate reductase gene variant as a susceptibility gene for non-syndromic conotruncal heart defects in an indian population. *Pediatr Cardiol* **2015**, *36*, 1470-1475. DOI: 10.1007/s00246-015-1188-3. [PubMed]
49. Pishva, S.R.; Vasudevan, R.; Etemad, A.; Heidari, F.; Komara, M.; Ismail, P.; Othman, F.; Karimi, A.; Sabri, M.R. Analysis of mthfr and mtrr gene polymorphisms in iranian ventricular septal defect subjects. *Int J Mol Sci* **2013**, *14*, 2739-2752. DOI: 10.3390/ijms14022739. [PubMed]
50. Sahiner, U.M.; Alanay, Y.; Alehan, D.; Tuncbilek, E.; Alikasifoglu, M. Methylene tetrahydrofolate reductase polymorphisms and homocysteine level in heart defects. *Pediatr Int* **2014**, *56*, 167-172. DOI: 10.1111/ped.12222. [PubMed]
51. Kocakap, B.D.S.; Sanli, C.; Cabuk, F.; Koc, M.; Kutsal, A. Association of MTHFR A1298C polymorphism with conotruncal heart disease. *Cardiol Young* **2015**, *25*, 1326-1331. DOI: 10.1017/S1047951114002467. [PubMed]
52. El-Abd, D.M.; Said, R.N.; Hanna, B.M.; El-naggar, N.F. Maternal and offspring methylenetetrahydrofolate reductase gene c677t polymorphism: Does it influence the prevalence of congenital heart defects in egyptian neonates? *Comp Clin Path* **2014**, *23*, 317-322. [CrossRef]
53. Elsayed, G.M.; Elsayed, S.M.; Ezz-Elarab, S.S. Maternal MTHFR C677T genotype and septal defects in offspring with down syndrome: A pilot study. *Egyptian Journal of Medical Human Genetics* **2014**, *15*, 39-44. [CrossRef]
54. Zidan, H.E.; Rezk, N.A.; Mohammed, D. MTHFR C677T and A1298C gene polymorphisms and their relation to homocysteine level in Egyptian childrenren with congenital heart diseases. *Gene* **2013**, *529*, 119-124. DOI: 10.1016/j.gene.2013. [PubMed] [CrossRef]
55. Kotby, A.; Anwar, M.; El-Masry, O.; Awady, M.; El-Nashar, A.; Meguid, N. Genetic variants in the methylenetetrahydrofolate reductase gene in egyptian childrenren with conotruncal heart defects and their mothers. *Maced J Med Sci* **2012**, *5*, 78-84. DOI: 10.3889/MJMS.1857-5773.2012.0222. [CrossRef]

Supplementary Table 2. Pooled analysis: MTHFR 677 genotype and the risk of congenital heart disease for all study groups (58 studies).

Genotype by ethnicity (number of studies)	Case (N=12,347) n (%)	Control (N=18,106) n (%)	Test of Heterogeneity			Statistical Model	Test of Association		
			Q	*p*	I^2 %		Risk Ratio	(95% CI)	*p*
TT (58)	1831 (14.8)	2170 (12.0)	113.6	< 0.0001	51.6	Random	1.30	(1.17 - 1.44)	< 0.0001
Caucasian (14)	710 (12.0)	1155 (11.6)	25.3	0.0211	48.6	Random	1.20	(1.01 - 1.42)	0.0417
East Asian (20)	756 (24.1)	548 (17.6)	46.4	0.0004	59	Random	1.41	(1.19 - 1.68)	< 0 .0001
South Asian (1)	0 (0.0)	0 (0.0)	-	-	-	-	-	-	-
Mixed (7)	131 (10.3)	155 (9.5)	2.08	0.9119	0	Fixed	1.01	(0.80 - 1.27)	0.9543
Mideast (3)	16 (4.8)	15 (4.8)	2.7	0.0989	63.3	Fixed	0.89	(0.43 - 1.81)	0.7388
Hispanic (5)	120 (19.2)	207 (15.6)	12.2	0.0160	67.2	Random	1.61	(0.98 - 2.65)	0.0598
African (8)	98 (10.2)	90 (5.5)	6.998	0.4290	0	Fixed	1.29	(1.00 - 1.66)	0.0510
CT (58)	5311 (43.0)	7653 (42.3)	76.0	0.0472	25	1.01	1.006	(0.98 - 1.03)	0.6431
Caucasian (14)	2693 (45.5)	4439 (44.4)	17.5	0.1774	25.7	Fixed	1.03	(0.99 - 1.07)	0.1109
East Asian (20)	1465 ()46.7	1454 (46.7)	23.1	0.2312	17.9	Fixed	0.98	(0.93 - 1.04)	0.4982
South Asian (1)	1 (1.0)	7 (7.8)	-	-	-	-	0.13	-	-
Mixed (7)	476 (37.5)	617 (37.8)	15.1	0.0192	60.4	Random	0.98	(0.82 - 1.17)	0.8538
Mideast (3)	146 (43.7)	137 (43.8)	1.04	0.5939	0	Fixed	1.01	(0.85 - 1.21)	0.8916
Hispanic (5)	274 (43.8)	604 (45.6)	1.9	0.7502	0	Fixed	0.93	(0.84 - 1.04)	0.1956
African (8)	256 (26.6)	395 (24.2)	8.5	0.2883	17.9	Fixed	1.03	(0.89 - 1.18)	0.7262
CC (58)	5205 (42.2)	8283 (45.7)	148.4	< 0 .0001	61.6	Random	0.91	(0.87 - 0.96)	0.0003
Caucasian (14)	2520 (42.5)	4404 (44.0)	24.3	0.0283	46.6	Random	0.94	(0.87 - 1.01)	0.0813
East Asian (20)	918 (29.2)	1111 (35.7)	56.2	< 0.0001	66.2	Random	0.81	(0.71 - 0.92)	0.0015
South Asian (1)	95 (99.0)	83 (92.2)	-	-	-	-	1.07	-	-
Mixed (7)	661 (52.1)	862 (52.8)	8.4	0.2071	29	Fixed	1.01	(0.94 - 1.09)	0.7709
Mideast (3)	172 (51.5)	161 (51.4)	1.9	0.3910	0	Fixed	1.00	(0.86 - 1.16)	0.9960
Hispanic (5)	232 (37.1)	515 (38.8)	4.4	0.3505	9.8	Fixed	1.01	(0.90 - 1.14)	0.8657
African (8)	607 (63.2)	1147 (70.3)	27.6	0.0003	74.7	Random	0.72	(0.55 - 0.95)	0.0204
TT+CT (58)	7142 (57.8)	9823 (54.3)	137.3	< 0.0001	58.5	Random	1.07	(1.04 - 1.11)	< 0.0001
Caucasian (14)	3403 (57.5)	5594 (56.0)	32.9	0.0018	60.4	Random	1.07	(1.01 - 1.14)	0.0253
East Asian (20)	2221 (70.8)	2002 (64.3)	50.2	0.0001	62.2	Random	1.10	(1.04 - 1.16)	0.0015
South Asian (1)	1 (1.0)	7 (7.8)	-	-	-	-	0.13	-	-
Mixed (7)	607 (47.9)	772 (47.2)	13.9	0.0306	56.9	Random	0.98	(0.86 - 1.13)	0.8135
Mideast (3)	162 (48.5)	152 (48.6)	1.8	0.3990	0	Fixed	1.00	(0.85 - 1.17)	0.9960
Hispanic (5)	394 (62.9)	811 (61.2)	5.4	0.2479	26	Fixed	0.99	(0.92 - 1.07)	0.8668
African (8)	354 (36.8)	485 (29.7)	18.7	0.0093	62.5	Random	1.24	(1.01 - 1.51)	0.0388
**Subgroups**									
TT risk >1 (11 countries)	5040 (53.4)	6162 (44.2)							
TT (42)	1069 (21.2)	885 (14.4)	75.4	0.0008	45.6	Random	1.43	(1.26 - 1.61)	< 0.0001
CT (42)	2311 (45.9)	2798 (45.4)	46.4	0.2601	11.6	Fixed	1.00	(0.96 - 1.04)	0.9429
CC (42)	1660 (32.9)	2479 (40.2)	84.5	< 0.0001	51.5	Random	0.84	(0.77 - 0.90)	< 0.0001
TT+CT (42)	3380 (67.1)	3683 (59.8)	78.6	0.0004	47.8	Random	1.10	(1.06 - 1.15)	< 0.0001
TT risk <1 (2 countries)	4119 (43.6)	7500 (53.8)							
TT (11)	431 (10.5)	812 (10.8)	6.9	0.7332	0	Fixed	0.97	(0.86 - 1.08)	0.5417
CT (11)	1579 (38.3)	2947 (39.3)	18.0	0.0552	44.4	Fixed	0.98	(0.93 - 1.03)	0.3946
CC (11)	2109 (51.2)	3741 (49.9)	16.3	0.0917	38.6	Fixed	1.02	(0.99 - 1.06)	0.2164
TT+CT (11)	2010 (48.8)	3759 (50.1)	30.9	0.0006	67.7	Random	1.01	(0.93 - 1.10)	0.8322
TT risk vary (3 countries)	281 (3.0)	276 (2.0)							
TT (4)	6 (2.1)	6 (2.2)	0.3	0.5603	0	Fixed	0.99	(0.34 - 2.90)	0.9801
CT (4)	83 (29.5)	92 (33.3)	7.5	0.0586	59.8	Fixed	0.91	(0.72 - 1.13)	0.3828
CC (4)	192 (68.3)	178 (64.5)	4.8	0.1857	37.7	Fixed	1.05	(0.94 - 1.17)	0.3808
TT+CT (4)	89 (31.7)	98 (35.5)	7.6	0.0558	60.4	Fixed	0.91	(0.74 - 1.12)	0.3795

Note. Q= Cochran's Q; CI=confidence interval. TT risk >1 (11 countries): Russia, Germany, Italy, Netherlands (5 studies), Portugal, Puerto Rico, Mexico (3 studies), Brazil (2 studies), Taiwan (2 studies), China (18 studies), and Egypt (7 studies); TT risk <1 (2 countries): US (9 studies), Turkey (2 studies); TT risk vary (3 countries): Austria, India, Iran; One study from 5 European countries.

Supplementary Table 3a Pooled analysis: *MTHFR* 677 genotypes and the risks of congenital heart disease for parents (23 Studies).

Genotypes by race or ethnicity (number of studies)	Case (N = 2596) n (%)	Control (N = 3056) n (%)	Test of Heterogeneity Q	*P*	I^2 %	Statistical Model	Test of Association Risk Ratio (95% CI)	*P*
**TT (23)**	429 (16.5)	379 (12.4)	27.0	0.21	18.6	Fixed	1.24 (1.09 -1.41)	0.0014
Caucasian (7)	105 (12.7)	150 (10.9)	8.4	0.2107	28.5	Fixed	1.13 (0.90 - 1.44)	0.2976
East Asian (7)	190 (21.6)	119 (15.5)	6.3	0.3909	4.7	Fixed	1.28 (1.04 - 1.58)	0.0226
Mixed (2)	70 (11.7)	38 (9.9)	0.7	0.4089	0	Fixed	1.18 (0.81 - 1.71)	0.3946
Hispanic (3)	32 (27.1)	40 (11.6)	3.9	0.1443	48.3	Fixed	1.94 (1.26 - 3.00)	0.0027
African (4)	32 (18.7)	32 (16.8)	3.3	0.3544	7.8	Fixed	1.05 (0.69 - 1.61)	0.8071
**CT (23)**	1131 (43.6)	1342 (43.9)	23.6	0.3667	6.9	Fixed	1.01 (0.95 - 1.07)	0.8559
Caucasian (7)	375 (45.3)	602 (43.9)	7.2	0.2997	17.1	Fixed	1.04 (0.94 - 1.15)	0.4344
East Asian (7)	400 (45.5)	346 (45.1)	4.0	0.6735	0	Fixed	0.96 (0.87 - 1.07)	0.4991
Mixed (2)	218 (36.3)	143 (37.4)	4.6	0.0318	78.3	Random	0.79 (0.44 - 1.43)	0.4418
Hispanic (3)	61 (51.7)	183 (53.2)	2.0	0.3686	0	Fixed	0.90 (0.72 - 1.13)	0.3713
African (4)	77 (45.0)	68 (35.8)	1.0	0.7991	0	Fixed	1.25 (0.97 - 1.62)	0.0830
**CC (23)**	1036 (39.9)	1335 (43.7)	20.7	0.5377	0	Fixed	0.92 (0.87 - 0.98)	0.0121
Caucasian (7)	347 (42.0)	620 (45.2)	5.8	0.4458	0	Fixed	0.93 (0.84 - 1.03)	0.1455
East Asian (7)	290 (33.0)	303 (39.5)	4.0	0.6764	0	Fixed	0.93 (0.82 - 1.05)	0.2211
Mixed (2)	312 (52.0)	201 (52.6)	2.4	0.1211	58.4	Fixed	0.99 (0.87 - 1.12)	0.8544
Hispanic (3)	25 (21.2)	121 (35.2)	1.6	0.4561	0	Fixed	0.75 (0.51 - 1.09)	0.1296
African (4)	62 (36.3)	90 (47.4)	4.0	0.2570	25.8	Fixed	0.79 (0.62 - 1.00)	0.0543
**TT + CT (23)**	1560 (60.1)	1721 (56.3)	22.1	0.4546	0.4	Fixed	1.06 (1.01 - 1.11)	0.0116
Caucasian (7)	480 (58.0)	752 (54.8)	6.7	0.3508	10.3	Fixed	1.06 (0.98 - 1.14)	0.1375
East Asian (7)	590 (67.0)	465 (60.5)	4.8	0.5735	0	Fixed	1.04 (0.97 - 1.12)	0.2273
Mixed (2)	288 (48.0)	181 (47.4)	3.0	0.0855	66.2	Fixed	1.01 (0.89 - 1.16)	0.8545
Hispanic (3)	93 (78.8)	223 (64.8)	1.2	0.5577	0	Fixed	1.12 (0.98 - 1.28)	0.1111
African (4)	109 (63.7)	100 (52.6)	3.1	0.3729	4	Fixed	1.19 (1.00 - 1.42)	0.0568
**Subgroups**								
TT risk >1 (9countries)								
TT (21)	423 (14.7)	373 (10.9)	26.5	0.1502	24.5	Fixed	1.24 (1.09 - 1.42)	0.0012
CT (21)	1109 (38.2)	1311 (40.0)	20.6	0.4237	2.7	Fixed	1.02 (0.95 - 1.08)	0.6480
CC (21)	1002 (47.1)	1311 (49.1)	15.8	0.7272	0	Fixed	0.91 (0.86 - 0.97)	0.0047
TT+CT (21)	1532 (52.9)	1684 (50.9)	17.5	0.618	0	Fixed	1.07 (1.02 - 1.12)	0.0045
TT risk vary (1 country)						Fixed		
TT (2)	6 (14.7)	6 (10.9)	0.3	0.5603	0	Fixed	0.99 (0.34 - 2.90)	0.9801
CT (2)	22 (38.2)	31 (40.0)	0.1	0.7052	0	Fixed	0.70 (0.46 - 1.06)	0.0919
CC (2)	34 (47.1)	24 (49.1)	0.9	0.3517	0	Fixed	1.39 (0.95 - 2.04)	0.0918
TT+CT (2)	28 (52.9)	37 (50.9)	0.5	0.4947	0	Fixed	0.75 (0.53 - 1.05)	0.0911

Note. Q: Cochran's Q; CI: confidence interval. TT risk >1 for 9 countries: Netherlands (3 studies), Russia, Italy, US, Puerto Rico, Mexico (2 studies), Brazil, China (7 studies), and Egypt (4 studies); TT risk vary: Austria (2 studies)

Supplementary Table 3b. Pooled analysis: *MTHFR* 677 genotype and the risk of congenital heart disease for mothers (19 studies).

Genotype by ethnicity (number of studies)	Case (N=2596) n (%)	Control (N=3056) n (%)	Test of Heterogeneity Q	p	I^2 %	Statistical Model	Test of Association Risk Ratio (95% Cl)	p
TT (19)	336 (16.5)	328 (12.8)	25.0	0.1240	28.1	Fixed	1.21 (1.04 - 1.39)	0.0106
Caucasian (5)	72 (12.7)	129 (11.8)	5.5	0.2391	27.4	Fixed	1.01 (0.77 - 1.31)	0.9704
East Asian (5)	130 (22.3)	89 (16.1)	5.7	0.2196	30.3	Fixed	1.30 (1.02 - 1.67)	0.0379
Mixed (2)	70 (11.7)	38 (9.9)	0.7	0.4089	0	Fixed	1.18 (0.81 - 1.71)	0.3946
Hispanic (3)	32 (27.1)	40 (11.6)	3.9	0.1443	48.3	Fixed	1.94 (1.26 – 3.00)	0.0027
African (4)	32 (18.7)	32 (16.8)	3.3	0.3544	7.8	Fixed	1.05 (0.69 - 1.61)	0.8071
CT (19)	881 (43.2)	1134 (44.3)	21.9	0.2368	17.8	Fixed	1.00 (0.94 - 1.08)	0.9249
Caucasian (5)	270 (47.6)	485 (44.5)	5.9	0.2048	32.5	Fixed	1.07 (0.95 - 1.20)	0.2689
East Asian (5)	255 (43.8)	255 (46.1)	1.9	0.7596	0	Fixed	0.92 (0.81 - 1.05)	0.2189
Mixed (2)	218 (36.3)	143 (37.4)	4.6	0.0318	78.3	Random	0.79 (0.44 - 1.43)	0.4418
Hispanic (3)	61 (51.7)	183 (53.2)	2.0	0.3686	0	Fixed	0.90 (0.72 - 1.13)	0.3713
African (4)	77 (45.0)	68 (35.8)	1.0	0.7991	0	Fixed	1.25 (0.97 - 1.62)	0.0830
CC (19)	821 (40.3)	1098 (42.9)	18.2	0.4395	1.4	Fixed	0.93 (0.87 - 1.00)	0.0498
Caucasian (5)	225 (39.7)	477 (43.7)	4.8	0.3117	16.1	Fixed	0.93 (0.82 - 1.06)	0.2557
East Asian (5)	197 (33.8)	209 (37.8)	2.6	0.6305	0	Fixed	0.97 (0.83 - 1.12)	0.6421
Mixed (2)	312 (52.0)	201 (52.6)	2.4	0.1211	58.4	Fixed	0.99 (0.87 - 1.12)	0.8544
Hispanic (3)	25 (21.2)	121 (35.2)	1.6	0.4561	0	Fixed	0.75 (0.51 - 1.09)	0.1296
African (4)	62 (36.3)	90 (47.4)	4.0	0.2570	25.8	Fixed	0.79 (0.62 - 1.00)	0.0543
TT+CT (19)	1217 (59.7)	1462 (57.1)	20.4	0.3105	11.8	Fixed	1.05 (1.00 - 1.11)	0.0477
Caucasian (5)	342 (60.3)	614 (56.3)	5.57	0.2335	28.2	Fixed	1.05 (0.97 - 1.15)	0.2431
East Asian (5)	385 (66.2)	344 (62.2)	3.25	0.5160	0	Fixed	1.02 (0.94 - 1.11)	0.6447
Mixed (2)	288 (48.0)	181 (47.4)	3.0	0.0855	66.2	Fixed	1.01 (0.89 - 1.16)	0.8545
Hispanic (3)	93 (78.8)	223 (64.8)	1.2	0.5577	0	Fixed	1.12 (0.98 - 1.26)	0.1111
African (4)	109 (63.7)	100 (52.6)	3.1	0.3729	4	Fixed	1.19 (1.00 - 1.42)	0.0568
**Subgroups**								
TT risk >1 (8 countries)	1619 (79.4)	2017 (78.8)						
TT (16)	293 (18.1)	265 (13.1)	18.2	0.2545	17.4	Fixed	1.29 (1.10 -1.51)	0.0014
CT (16)	685 (42.3)	906 (44.9)	14.6	0.4813	0	Fixed	0.97 (0.89 - 1.05)	0.5168
CC (16)	641 (39.6)	846 (41.9)	14.2	0.5091	0	Fixed	0.51 (0.86 - 1.01)	0.0777
TT+CT (16)	978 (60.4)	1171 (58.1)	16.3	0.3636	7.9	Fixed	1.05 (1.00 - 1.11)	0.0752
TT risk <1 (2 countries)	419 (20.6)	543 (21.2)						
TT (3)	43 (10.3)	63 (11.6)	2.9	0.2349	31	Fixed	0.89 (0.60 - 1.25)	0.4417
CT (3)	196 (46.8)	228 (42.0)	4.7	0.0964	57.2	Fixed	1.11 (0.96 - 1.28)	0.1734
CC (3)	180 (43.0)	252 (46.4)	4.0	0.0964	50.4	Fixed	1.11 (0.81 - 1.08)	0.3858
TT+CT (3)	239 (57.0)	291 (53.6)	4.1	0.1276	51.4	Fixed	1.05 (0.94 - 1.18)	0.3802

Note. Q: Cochran's Q; CI: confidence interval. TT risk >1 (8 countries): Russia, Italy, US, Puerto Rico, Mexico (2 studies), Brazil, China (5 studies), and Egypt (4 studies); TT risk <1 (2 countries): Netherlands (2 studies) and Austria.

Supplementary Table 3c. Pooled analysis: *MTHFR* 677 genotype and the risk of congenital heart disease for fathers (4 studies).

Genotype by ethnicity (number of studies)	Case (N=558) n (%)	Control (N=496) n (%)	Test of Heterogeneity Q	p	I^2 %	Statistical Model	Test of Association Risk Ratio (95% Cl)	p
TT (4)	93 (16.7)	51 (10.3)	1.5	0.6737	0	Fixed	1.39 (1.02 - 1.91)	0.0395
Caucasian (2)	33 (12.7)	21 (7.5)	0.0	0.8484	0	Fixed	1.70 (1.01 - 2.86)	0.0450
East Asian (2)	60 (20.1)	30 (14.0)	0.6	0.4544	0	Fixed	1.22 (0.82 - 1.81)	0.3257
CT (4)	250 (44.8)	208 (41.9)	1.7	0.6316	0	Fixed	1.02 (0.88 - 1.17)	0.8280
Caucasian (2)	105 (40.4)	117 (41.6)	0.7	0.4056	0	Fixed	0.97 (0.79 - 1.19)	0.7675
East Asian (2)	145 (48.7)	91 (42.3)	0.8	0.3748	0	Fixed	1.06 (0.88 - 1.28)	0.5257
CC (4)	215 (38.5)	237 (47.8)	2.3	0.5072	0	Fixed	0.89 (0.78 - 1.02)	0.0893
Caucasian (2)	122 (46.9)	143 (50.9)	1.0	0.3098	3.1	Fixed	0.92 (0.77 - 1.10)	0.3553
East Asian (2)	93 (22.5)	94 (43.7)	1.2	0.0099	19.5	Random	0.86 (0.67 - 1.11)	0.2490
TT+CT (4)	343 (61.5)	259 (52.2)	1.1	0.7813	0	Fixed	1.09 (0.99 - 1.21)	0.0920
Caucasian (2)	138 (53.1)	138 (49.1)	1.0	0.3204	0	Fixed	1.08 (0.92 - 1.28)	0.3530
East Asian (2)	205 (68.8)	121 (56.3)	0.1	0.7383	0	Fixed	1.10 (0.97 - 1.25)	0.1301

Note. Q: Cochran's Q; CI: confidence interval. *TT* risk >1 for all countries (Austria, China (2 studies), and Netherlands) of fathers. All mixed CHD.

Supplementary Table 4a. Pooled analysis: *MTHFR* C677T polymorphism and the risk of congenital heart disease (CHD) per CHD types for children (35 study groups).

Genotype by disease type and ethnicity (number of studies)	Case (N=9751) n (%)	Control (N=15,050) n (%)	Test of Heterogeneity Q	*p*	I^2 %	Statistical Model	Test of Association Risk Ratio (95% Cl)	*p*
TT (35)	1402 (14.4)	1791 (11.9)	85.2	< 0.0001	62.4	Random	1.30 (1.14 - 1.48)	0.0001
Mixed CHD (23)	1179 (14.0)	1571 (12.1)	62.3	< 0 .0001	64.7	Random	1.25 (1.08 - 1.45)	0.0028
Caucasian (5)	589 (11.8)	959 (11.9)	8.8	0.0660	54.6	Fixed	1.01 (0.91 - 1.11)	0.9164
East Asian (9)	404 (23.3)	341 (18.4)	34.7	< 0.0001	76.9	Random	1.37 (1.037 - 1.82)	0.0300
Mixed (3)	22 (7.3)	41 (6.8)	0.3	0.8638	0	Fixed	0.89 (0.54 - 1.47)	0.6451
Mideast (1)	14 (10.3)	7 (7.5)	-	-	-	-	1.37 -	-
Hispanic (2)	88 (17.3)	167 (17.0)	1.7	0.1923	41.2	Fixed	1.02 (0.81 - 1.30)	0.8420
African (3)	62 (8.2)	56 (4.0)	3.47	0.1764	42.4	Fixed	1.42 (1.02 - 1.96)	0.0371
Cyanotic CHD (9)	211 (18.9)	185 (11.7)	14.7	0.0397	52.5	Random	1.41 (1.03 - 1.94)	0.0311
Caucasian (1)	6 (15.8)	14 (5.6)	-	-	-	-	2.82 -	-
East Asian (3)	160 (31.4)	85 (18.6)	1.2	0.5620	0	Fixed	1.73 (1.37 - 2.19)	< 0.0001
South Asian (1)	0 (0.0)	0 (0.0)	-	-	-	-	1.00 -	-
Mixed (2)	39 (10.6)	76 (11.7)	0.1	0.7720	0	Fixed	0.92 (0.63 - 1.34)	0.6700
Mideast (1)	2 (2.7)	8 (8.4)	-	-	-	-	0.32 -	-
African (1)	4 (13.3)	2 (6.7)	-	-	-	-	1.99 -	-
Acyanotic CHD (3)	12 (6.2)	35 (7.6)	0.1	0.7900	0	Fixed	1.64 (0.90 - 3.01)	0.1092
Caucasian (1)	10 (18.2)	32 (10.7)	-	-	-	-	1.70 -	-
East Asian (1)	2 (11.8)	3 (8.8)	-	-	-	-	1.34 -	-
Mideast (1)	0 (0.0)	0 (0.0)	-	-	-	-	1.00 -	-
CT (35)	4180 (42.9)	6311 (41.9)	52.3	0.0023	35.0	Random	1.00 (0.96 - 1.05)	0.9822
Mixed CHD (23)	3635 (43.1)	5420 (41.6)	36.1	0.0299	39.0	Random	1.00 (0.95 - 1.05)	0.9984
Caucasian (5)	2265 (45.3)	3579 (44.3)	5.7	0.2212		Fixed	1.02 (0.98 - 1.06)	0.2609
East Asian (9)	838 (48.4)	833 (47.7)	15.2	0.0107	47.7	Fixed	1.01 (0.95 - 1.19)	0.6997
Mixed (3)	101 (33.6)	179 (29.8)	10.1	0.0063	80.3	Random	1.04 (0.85 - 1.27)	0.7397
Mideast (1)	53 (39.0)	39 (41.9)	-	-	-	-	0.93 -	-
Hispanic (2)	213 (41.9)	421 (42.9)	0.0	0.8641	0	Fixed	0.94 (0.83 - 1.06)	0.3174
African (3)	165 (21.7)	319 (22.6)	1.9	0.3806	0	Fixed	0.93 (0.79 - 1.11)	0.4302
Cyanotic CHD (9)	447 (40.1)	691 (43.8)	8.9	0.3510	10.1	Fixed	0.94 (0.86 - 1.03)	0.1915
Caucasian (1)	20 (52.6)	124 (49.4)	-	-	-	-	1.07 -	-
East Asian (3)	222 (43.6)	213 (46.5)	1.7	0.4256	0	Fixed	0.90 (0.78 - 1.03)	0.1195
South Asian (1)	1 (1.0)	7 (7.8)	-	-	-	-	0.13 -	-
Mixed (2)	157 (42.8)	295 (45.2)	0.0	0.8926	0	Fixed	0.96 (0.83 - 1.12)	0.6338
Mideast (1)	33 (44.0)	44 (46.3)	-	-	-	-	0.95 -	-
African (1)	14 (46.7)	8 (26.7)	-	-	-	-	1.75 -	-
Acyanotic CHD (3)	98 (50.3)	200 (43.6)	1.7	0.4302	0	Fixed	1.19 (0.99 - 1.43)	0.0586
Caucasian (1)	33 (60.0)	134 (44.7)	-	-	-	-	1.34 -	-
East Asian (1)	5 (29.4)	12 (35.3)	-	-	-	-	0.83 -	-
Mideast (1)	60 (48.8)	54 (43.2)	-	-	-	-	1.13 -	-
CC (35)	4169 (42.8)	6948 (46.2)	124.0	< 0.0001	72.6	Random	0.90 (0.85 - 0.96)	0.0014
Mixed CHD (23)	3627 (43.0)	6024 (46.3)	85.8	< 0.0001	74.4	Random	0.90 (0.83 - 0.97)	0.0165
Caucasian (5)	2149 (43.0)	3537 (43.8)	9. 3	0.0549	56.8	Fixed	0.98 (0.94 - 1.02)	0.2329
East Asian (9)	491 (28.3)	629 (33.9)	44.8	< 0.0001	82.2	Random	0.73 (0.57 - 0.93)	0.0125
Mixed (3)	178 (59.1)	380 (63.3)	5.2	0.0742	61.6	Fixed	1.00 (0.89 - 1.12)	0.9443
Mideast (1)	69 (50.7)	47 (50.5)	-	-	-	-	1.00 -	-
Hispanic (2)	207 (40.7)	394 (40.1)	0.4	0.5497	0	Fixed	1.06 (0.93 - 1.79)	0.1288
African (3)	533 (70.1)	1037 (73.4)	14.7	0.0007	86.4	Random	0.64 (0.34 - 1.22)	0.1729
Cyanotic CHD (9)	457 (41.0)	700 (44.4)	33.9	< 0.0001	76.4	Random	0.90 (0.76 - 1.07)	0.2300
Caucasian (1)	12 (31.6)	113 (45.0)	-	-	-	-	0.70 -	-
East Asian (3)	127 (25.0)	160 (34.9)	4.1	0.1288	51.2	Fixed	0.76 (0.63 - 0.92)	0.0050
South Asian (1)	95 (99.0)	83 (92.2)	-	-	-	-	1.07 -	-
Mixed (2)	171 (46.6)	281 (43.1)	0.1	0.7066	0	Fixed	1.06 (0.92 - 1.22)	0.4499
Mideast (1)	40 (53.3)	43 (45.3)	-	-	-	-	1.18 -	-
African (1)	12 (40.0)	20 (66.7)	-	-	-	-	0.60 -	-
Acyanotic CHD (3)	85 (43.6)	224 (48.8)	6.1	0.0476	67.2	Random	0.79 (0.53 - 1.20)	0.2716
Caucasian (1)	12 (21.8)	134 (44.7)	-	-	-	-	0.49 -	-
East Asian (1)	10 (58.8)	19 (55.9)	-	-	-	-	1.05 -	-

Genotype by disease type and ethnicity (number of studies)	Case (N=9751) n (%)	Control (N=15,050) n (%)	Test of Heterogeneity Q	p	I^2 %	Statistical Model	Test of Association Risk Ratio (95% CI)		p
Mideast (1)	63 (51.2)	71 (56.8)	-	-	-	-	0.90	-	-
TT + CT (35)	5582 (57.2)	8102 (53.8)	115.6	< 0.0001	70.6	Random	1.09	(1.04 - 1.14)	0.0008
Mixed CHDs (23)	4814 (57.0)	6991 (53.7)	84.7	< 0.0001	74	Random	1.08	(1.02 - 1.14)	0.0077
Caucasian (5)	2854 (57.0)	4538 (56.2)	10.1	0.0380	60.6	Random	1.03	(0.96 - 1.10)	0.4416
East Asian (9)	1242 (71.7)	1224 (66.1)	40.7	< 0.0001	80.3	Random	1.13	(1.02 - 1.25)	0.0159
Mixed (3)	123 (40.9)	220 (36.7)	10.5	0.0054	80.9	Random	1.26	(0.73 - 2.15)	0.4044
Mideast (1)	67 (49.3)	46 (49.5)	-	-	-	-	1.00	-	-
Hispanic (2)	301 (59.3)	588 (59.9)	1.1	0.3013	6.4	Fixed	0.96	(0.88 - 1.05)	0.3953
African (3)	227 (29.9)	375 (26.6)	12.1	0.0024	83.4	Random	1.25	(0.84 - 1.87)	0.2671
Cyanotic CHDs (9)	658 (59.0)	876 (55.6)	17.7	0.0235	54.8	Random	1.07	(0.96 - 1.19)	0.2205
Caucasian (1)	26 (68.4)	138 (55.0)	-	-	-	-	1.24	-	-
East Asian (3)	382 (75.0)	298 (65.1)	1.3	0.5346	0	Fixed	1.13	(1.04 - 1.23)	0.0061
South Asian (1)	1 (1.0)	7 (7.8)	-	-	-	-	0.13	-	-
Mixed (2)	196 (53.4)	371 (56.9)	0.1	0.7534	0	Fixed	0.96	(0.85 - 1.08)	0.4536
Mideast (1)	35 (46.7)	52 (54.7)	-	-	-	-	0.85	-	-
African (1)	18 (60.0)	10 (33.3)	-	-	-	-	1.80	-	-
Acyanotic CHDs (3)	110 (56.4)	235 (51.2)	3.3	0.1887	40	Fixed	1.24	(1.06 - 1.45)	0.0066
Caucasian (1)	43 (78.2)	166 (55.3)	-	-	-	-	1.41	-	-
East Asian (1)	7 (41.2)	15 (44.1)	-	-	-	-	0.93	-	-
Mideast (1)	60 (56.4)	54 (43.2)	-	-	-	-	1.31	-	-

Note. Q: Cochran's Q; CI: confidence interval.

Supplementary Table 4b. Pooled analysis: *MTHFR* C677T polymorphism and the risk of congenital heart disease (CHD) per CHD types for parents (23 studies).

Genotype by disease type and ethnicity (number of studies)	Case (N=2596) n (%)	Control (N=3056) n (%)	Test of Heterogeneity Q	*p*	I^2 %	Statistical Model	Test of Association Risk Ratio (95% CI)	*p*
**TT (23)**	429 (16.5)	379 (12.4)	27.0	0.21	18.6	Fixed	1.24 (1.09 -1.41)	0.0014
Mixed CHDs (19)	392 (16.4)	324 (12.0)	18.2	0.4401	1.3	Fixed	1.23 (1.07 -1.42)	0.0032
Caucasian (6)	82 (11.3)	110 (9.4)	8.4	0.1364	40.3	Fixed	1.14 (0.86 - 1.50)	0.3539
East Asian (7)	190 (21.6)	119 (15.5)	6.3	0.3909	4.7	Fixed	1.28 (1.04 - 1.58)	0.0226
Mixed (2)	70 (11.7)	38 (9.9)	0.7	0.4089	0	Fixed	1.18 (0.81 - 1.71)	0.3946
Hispanic (2)	20 (23.0)	33 (11.7)	1.5	0.2218	33	Fixed	1.49 (0.90 - 2.56)	0.1159
African (2)	30 (28.3)	24 (24.5)	0.2	0.6435	0	Fixed	1.24 (0.80 - 1.93)	0.3409
Cyanotic CHDs (3)	37 (22.6)	48 (16.4)	5.5	0.0632	63.8	Fixed	1.47 (1.01 - 2.16)	0.0470
Caucasian (1)	23 (22.3)	40 (20.0)	-	-	-	-	1.12 -	-
Hispanic (1)	12 (38.7)	7 (11.3)	-	-	-	-	3.43 -	-
African (1)	2 (6.7)	1 (3.2)	-	-	-	-	2.09 -	-
Acyanotic CHDs (1) African (1)	0 (0.0)	7 (11.5)	-	-	-	-	- -	-
**CT (23)**	1131 (43.6)	1342 (43.9)	23.6	0.3667	6.9	Fixed	1.01 (0.95 - 1.07)	0.8559
Mixed CHDs (19)	1034 (43.1)	1169 (43.3)	6.5	0.2597	23.2	Fixed	1.06 (0.95 - 1.18)	0.3077
Caucasian (6)	322 (44.5)	494 (42.2)	19.5	0.3620	7.7	Fixed	1.01 (0.94 - 1.07)	0.8748
East Asian (7)	400 (45.5)	346 (45.1)	4.0	0.6735	0	Fixed	0.96 (0.87 - 1.07)	0.4991
Mixed (2)	218 (36.3)	143 (37.4)	4.6	0.0318	78.3	Random	0.79 (0.44 - 1.43)	0.4418
Hispanic (2)	49 (56.3)	152 (53.9)	1.3	0.2561	22.5	Fixed	0.95 (0.74 - 1.21)	0.6617
African (2)	45 (42.5)	34 (34.7)	0.0	0.9131	0	Fixed	1.19 (0.84 - 1.68)	0.3346
Cyanotic (3)	81 (49.4)	149 (50.9)	3.7	0.1564	46.1	Fixed	0.98 (0.81 - 1.20)	0.8693
Caucasian (1)	53 (51.5)	108 (54.0)	-	-	-	-	0.95 -	-
Hispanic (1)	12 (38.7)	31 (50.0)	-	-	-	-	0.77 -	-
African (1)	16 (53.3)	10 (32.3)	-	-	-	-	1.65 -	-
Acyanotic (1) African (1)	16 (45.7)	24 (39.3)	-	-	-	-	1.16 -	-
**CC (23)**	1036 (39.9)	1335 (43.7)	20.7	0.5377	0	Fixed	0.92 (0.87 - 0.98)	0.0121
Mixed CHD (19)	971 (40.5)	1209 (44.7)	15.8	0.6036	0	Fixed	0.93 (0.87 – 1.00)	0.0225
Caucasian (6)	320 (44.2)	568 (48.5)	5.6	0.3463	10.8	Fixed	0.92 (0.83 - 1.02)	0.1191
East Asian (7)	290 (33.0)	303 (39.5)	4.0	0.6764	0	Fixed	0.93 (0.82 - 1.05)	0.2211
Mixed (2)	312 (52.0)	201 (52.6)	2.4	0.1211	58.4	Fixed	0.99 (0.87 - 1.12)	0.8544
Hispanic (2)	18 (20.7)	97 (34.4)	0.8	0.3830	0	Fixed	0.83 (0.54 - 1.29)	0.4145
African (2)	31 (29.2)	40 (40.8)	0.1	0.7578	0	Fixed	0.70 (0.48 - 1.03)	0.0675
Cyanotic (3)	46 (28.0)	96 (32.8)	3.0	0.2245	33.1	Fixed	0.81 (0.60 - 1.08)	0.1427
Caucasian (1)	27 (26.2)	52 (26.0)	-	-	-	-	1.01 -	-
Hispanic (1)	7 (22.6)	24 (38.7)	-	-	-	-	0.58 -	-
African (1)	12 (40.0)	20 (64.5)	-	-	-	-	0.62 -	-
Acyanotic (1) African (1)	19 (54.3)	30 (49.2)	-	-	-	-	1.10 -	-
**TT + CT (23)**	1560 (60.1)	1721 (56.3)	22.1	0.4546	0.4	Fixed	1.06 (1.01 - 1.11)	0.0116
Mixed (19)	1426 (59.5)	1493 (55.3)	16.5	0.5553	0	Fixed	1.06 (1.01 - 1.11)	0.0219
Caucasian (6)	404 (55.8)	604 (51.5)	5.8	0.3224	14.3	Fixed	1.08 (0.98 - 1.17)	0.1107
Mixed (2)	348 (58.6)	232 (53.5)	3.0	0.0855	66.2	Fixed	1.01 (0.89 - 1.16)	0.8545
Hispanic (2)	119 (68.0)	190 (63.8)	0.2	0.6563	0	Fixed	1.07 (0.92 - 1.24)	0.4080
East Asian (7)	590 (67.0)	465 (60.5)	4.8	0.5735	0	Fixed	1.04 (0.97 - 1.12)	0.2273
African (2)	75 (70.8)	58 (59.2)	0.0	0.9419	0	Fixed	1.21 (0.99 - 1.48)	0.0691
Cyanotic (3)	118 (72.0)	197 (67.3)	5.1	0.0769	61	Fixed	1.10 (0.97 - 1.25)	0.1319
Caucasian (1)	76 (73.8)	148 (74.0)	-	-	-	-	1.00 -	-
Hispanic (1)	24 (77.4)	38 (61.3)	-	-	-	-	1.26 -	-
African (1)	18 (60.0)	11 (35.5)	-	-	-	-	1.69 -	-
Acyanotic (1) African (1)	16 (45.7)	31 (50.8)	-	-	-	-	0.90 -	-

Note. Q: Cochran's Q; CI: confidence interval.

Supplementary Table 4c. Pooled analysis: *MTHFR* C677T polymorphism and the risk of congenital heart disease (CHD) per CHD types for mothers (19 studies).

Genotype by disease type and ethnicity (number of studies)	Case (N=2038) n (%)	Control (N=2560) n (%)	Test of Heterogeneity Q	p	I^2 %	Statistical Model	Test of Association Risk Ratio (95% CI)	p
TT (19)	336 (16.5)	328 (12.8)	25.0	0.1240	28.1	Fixed	1.21 (1.04 - 1.39)	0.0106
Mixed CHDs (15)	299 (16.3)	273 (12.4)	16.1	0.3101	12.8	Fixed	1.20 (1.02 - 1.40)	0.0242
Caucasian (4)	49 (10.6)	89 (10.0)	5.3	0.1511	43.4	Fixed	0.96 (0.69 - 1.33)	0.7894
East Asian (5)	130 (22.3)	89 (16.1)	5.7	0.2196	30.3	Fixed	1.30 (1.02 - 1.67)	0.0379
Mixed (2)	70 (11.7)	38 (9.9)	0.7	0.4089	0	Fixed	1.18 (0.81 - 1.71)	0.3946
Hispanic (2)	20 (23.0)	33 (11.7)	1.5	0.2218	33	Fixed	1.49 (0.90 - 2.56)	0.1159
African (2)	30 (28.3)	24 (24.5)	0.2	0.6435	0	Fixed	1.24 (0.80 - 1.93)	0.3409
Cyanotic CHDs (3)	37 (22.6)	48 (16.4)	5.5	0.0632	63.8	Fixed	1.47 (1.01 - 2.16)	0.0470
Caucasian (1)	23 (22.3)	40 (20.0)	-	-	-	-	1.12 -	-
Hispanic (1)	12 (38.7)	7 (11.3)	-	-	-	-	3.43 -	-
African (1)	2 (6.7)	1 (3.2)	-	-	-	-	2.09 -	-
Acyanotic CHDs (1) African (1)	0 (0.0)	7 (11.5)	-	-	-	-	- -	-
CT (19)	881 (43.2)	1134 (44.3)	21.9	0.2368	17.8	Fixed	1.00 (0.94 - 1.08)	0.9249
Mixed CHDs (15)	784 (42.6)	961 (43.6)	17.8	0.2184	21.1	Fixed	1.00 (0.93 - 1.08)	0.9500
Caucasian (4)	217 (46.8)	377 (42.3)	4.7	0.1962	36	Fixed	1.10 (0.97 - 1.26)	0.1468
East Asian (5)	255 (43.8)	255 (46.1)	1.9	0.7596	0	Fixed	0.92 (0.81 - 1.05)	0.2189
Mixed (2)	218 (36.3)	143 (37.4)	4.6	0.0318	78.3	Random	0.79 (0.44 - 1.43)	0.4418
Hispanic (2)	49 (56.3)	152 (53.9)	1.3	0.2561	22.5	Fixed	0.95 (0.74 - 1.21)	0.6617
African (2)	45 (42.5)	34 (34.7)	0.0	0.9131	0	Fixed	1.19 (0.84 - 1.68)	0.3346
Cyanotic (3)	81 (49.4)	149 (50.9)	3.7	0.1564	46.1	Fixed	0.98 (0.81 - 1.20)	0.8693
Caucasian (1)	53 (51.5)	108 (54.0)	-	-	-	-	0.95 -	-
Hispanic (1)	12 (38.7)	31 (50.0)	-	-	-	-	0.77 -	-
African (1)	16 (53.3)	10 (32.3)	-	-	-	-	1.65 -	-
Acyanotic (1) African (1)	16 (45.7)	24 (39.3)	-	-	-	-	1.16 -	-
CC (19)	821 (40.3)	1098 (42.9)	18.2	0.4395	1.4	Fixed	0.93 (0.87 - 1.00)	0.0498
Mixed CHD (15)	756 (41.1)	972 (44.1)	13.3	0.5020	0	Fixed	0.94 (0.87 - 1.01)	0.0918
Caucasian (4)	198 (42.7)	425 (47.7)	4.6	0.2059	34.4	Fixed	0.92 (0.80 - 1.05)	0.2093
East Asian (5)	197 (33.8)	209 (37.8)	2.6	0.6305	0	Fixed	0.97 (0.83 - 1.12)	0.6421
Mixed (2)	312 (52.0)	201 (52.6)	2.4	0.1211	58.4	Fixed	0.99 (0.87 - 1.12)	0.8544
Hispanic (2)	18 (20.7)	97 (34.4)	0.8	0.3830	0	Fixed	0.83 (0.58 - 1.29)	0.4145
African (2)	31 (29.2)	40 (40.8)	0.1	0.7578	0	Fixed	0.70 (0.48 - 1.03)	0.0675
Cyanotic (3)	46 (28.0)	96 (32.8)	3.0	0.2245	33.1	Fixed	0.81 (0.60 - 1.08)	0.1427
Caucasian (1)	27 (26.2)	52 (26.0)	-	-	-	-	1.01 -	-
Hispanic (1)	7 (22.6)	24 (38.7)	-	-	-	-	0.58 -	-
African (1)	12 (40.0)	20 (64.5)	-	-	-	-	0.62 -	-
Acyanotic (1) African (1)	19 (54.3)	30 (49.2)	-	-	-	-	1.10 -	-
TT + CT (19)	1217 (59.7)	1462 (57.1)	20.4	0.3105	11.8	Fixed	1.05 (1.00 - 1.12)	0.0477
Mixed (15)	1083 (58.9)	1234 (56.0)	14.8	0.3917	5.4	Fixed	1.05 (0.99 - 1.11)	0.0891
Caucasian (4)	266 (57.4)	466 (52.3)	4.8	0.1834	38.1	Fixed	1.07 (0.97 - 1.19)	0.1948
Mixed (2)	348 (58.6)	232 (53.5)	3.0	0.0855	66.2	Fixed	1.01 (0.89 - 1.16)	0.8545
Hispanic (2)	119 (68.0)	190 (63.8)	0.198	0.6563	0	Fixed	1.07 (0.92 - 1.24)	0.4080
East Asian (5)	275 (66.8)	288 (57.8)	3.3	0.5160	0	Fixed	1.02 (0.94 - 1.11)	0.6447
African (2)	75 (70.8)	58 (59.2)	0.0	0.9419	0	Fixed	1.21 (0.99 - 1.48)	0.0691
Cyanotic (3)	118 (72.0)	197 (67.3)	5.1	0.0769	61	Fixed	1.10 (0.97 - 1.25)	0.1319
Caucasian (1)	76 (73.8)	148 (74.0)	-	-	-	-	1.00 -	-
Hispanic (1)	24 (77.4)	38 (61.3)	-	-	-	-	1.26 -	-
African (1)	18 (60.0)	11 (35.5)	-	-	-	-	1.69 -	-
Acyanotic (1) African (1)	16 (45.7)	31 (50.8)	-	-	-	-	0.90 -	-

Note. Q: Cochran's Q; CI: confidence interval.

Supplementary Table 5a. Pooled analysis: *MTHFR* 1298 genotype and the risk of congenital heart disease for all study groups (21 studies).

Genotype by ethnicity (number of studies)	Case (N=2754) n (%)	Control (N=3419) n (%)	Test of Heterogeneity Q	*p*	I2 %	Statistical Model	Test of Association Risk Ratio (95% CI)	*p*
CC (21)	333 (12.1)	287 (8.4)	55.7	< 0 .0001	65.9	Random	1.44 (1.07 - 1.95)	0.0174
Caucasian (5)	80 (9.5)	148 (11.3)	6.1	0.1949	34	Fixed	0.81 (0.62 - 1.07)	0.1345
East Asian (7)	54 (4.2)	44 (2.9)	10.9	0.0532	54.2	Fixed	1.52 (1.03 - 2.22)	0.0330
South Asian (1)	37 (38.5)	22 (22.0)	-	-	-	-	1.75 -	-
Mixed (2)	5 (4.8)	4 (6.3)	2.1	0.1444	53.1	Fixed	0.75 (0.20 - 2.79)	0.6642
Mideast (2)	37 (18.0)	19 (9.9)	0.1	0.7335	0	Fixed	1.87 (1.10 - 3.18)	0.0208
African (4)	120 (54.5)	50 (22.7)	18.8	0.0003	84.1	Random	3.34 (1.40 - 7.94)	0.0064
AC (21)	988 (35.9)	1225 (35.8)	73.7	< 0.0001	72.9	Random	1.02 (0.88 - 1.18)	0.8059
Caucasian (5)	349 (41.6)	594 (45.2)	8.3	0.0821	51.7	Fixed	0.89 (0.80 - 0.99)	0.0320
East Asian (7)	398 (30.9)	413 (27.0)	31.6	< 0.0001	81	Random	1.21 (0.88 - 1.65)	0.2403
South Asian (1)	32 (33.3)	20 (20.0)	-	-	-	-	1.67 -	-
Mixed (2)	38 (36.5)	26 (40.6)	0.03	0.8592	0	Fixed	0.90 (0.61 - 1.33)	0.6019
Mideast (2)	104 (50.5)	91 (47.2)	5.3	0.0208	81.3	Random	1.08 (0.67 - 1.74)	0.7659
African (4)	67 (30.5)	81 (36.8)	15.4	0.0015	80.5	Random	0.64 (0.31 - 1.29)	0.2115
AA (21)	1433 (52.0)	1907 (55.8)	111.2	< 0.0001	82	Random	0.91 (0.81 - 1.024)	0.1172
Caucasian (5)	409 (48.8)	573 (43.6)	12.9	0.0120	68.9	Random	1.19 (1.00 - 1.41)	0.0526
East Asian (7)	838 (65.0)	1071 (70.1)	27.6	0.0001	78.3	Random	0.92 (0.82 - 1.04)	0.1724
South Asian (1)	27 (28.1)	58 (58.0)	-	-	-	-	0.48 -	-
Mixed (2)	61 (58.7)	34 (53.1)	0.74	0.3891	0	Fixed	1.10 (0.84 - 1.46)	0.4867
Mideast (2)	65 (31.6)	82 (42.7)	3.9	0.0495	74.1	Fixed	0.75 (0.43 - 1.30)	0.3053
African (4)	33 (15.0)	89 (40.5)	4.95	0.1755	39.4	Fixed	0.37 (0.26 - 0.53)	< 0.0001
CC + AC (21)	1321 (48.0)	1512 (44.2)	107.3	< 0.0001	81.4	Random	1.16 (1.02 - 1.32)	0.0293
Caucasian (5)	429 (51.2)	742 (56.4)	10.8	0.0295	62.8	Random	0.88 (0.76 - 1.02)	0.0848
East Asian (7)	452 (35.0)	457 (29.9)	31.1	< 0.0001	80.7	Random	1.24 (0.94 -1.64)	0.1361
South Asian (1)	69 (71.9)	42 (42.0)	-	-	-	-	1.71	
Mixed (2)	43 (41.3)	30 (46.9)	0.7	0.3899	0	Fixed	0.88 (0.62 - 1.25)	0.4777
Mideast (2)	141 (68.4)	110 (57.3)	5.5	0.0188	81.9	Random	1.20 (0.83 - 1.74)	0.3261
African (4)	187 (85.0)	131 (59.5)	2.7	0.4343	0	Fixed	1.43 (1.26 -1.61)	< 0.0001
**Subgroups**								
CC risk >1 (6 countries)	1915 (69.5)	2240 (65.5)						
CC (15)	256 (13.4)	148 (6.6)	29.8	0.0050	56.4	Random	1.85 (1.34 - 2.54)	0.0002
AC (15)	647 (33.8)	691 (30.8)	57.6	< 0.0001	75.7	Random	1.09 (0.89 - 1.33)	0.4025
AA (15)	1012 (52.8)	1401 (62.5)	72.6	< 0.0001	80.7	Random	0.80 (0.70 - 0.92)	0.0014
CC + AC (15)	903 (47.2)	839 (37.5)	54.2	< 0.0001	74.2	Random	1.31 (1.15 - 1.51)	< 0.0001
CC risk <1 (3 countries)	839 (30.5)	1179 (34.5)						
CC (6)	77 (9.2)	139 (11.8)	7.4	0.1904	32.7	Fixed	0.78 (0.59 -1.03)	0.0780
AC (6)	341 (40.6)	534 (45.3)	7.0	0.2201	28.6	Fixed	0.87 (0.78 - 0.97)	0.0135
AA (6)	421 (50.2)	506 (42.9)	10.2	0.0708	50.8	Fixed	1.20 (1.09 - 1.33)	0.0003
CC + AC (6)	418 (49.8)	673 (57.1)	8.9	0.1147	43.6	Fixed	0.85 (0.78 - 0.93)	0.0004

Note. Q: Cochran's Q; CI: confidence interval. CC risk >1 (6 countries): Italy, Taiwan, China (6 studies), India, Turkey (2 studies), and Egypt (4 studies); CC risk <1 (3 countries): Russian, Netherlands (3 studies), and Brazil (2 studies).

Supplementary Table 5b. Pooled analysis: *MTHFR* 1298 genotype and the risk of congenital heart disease for children (13 studies).

Genotype by ethnicity (number of studies)	Case (N=1835) n (%)	Control (N=2003) n (%)	Test of Heterogeneity Q	p	I^2 %	Statistical Model	Test of Association Risk Ratio (95% CI)	p
CC (13)	198 (10.8)	133 (6.6)	19.3	0.0564	42.9	Fixed	1.56 (1.28 - 1.91)	< 0.0001
Caucasian (1)	27 (11.8)	25 (10.0)	-	-	-	-	1.18 -	-
East Asian (6)	35 (3.1)	38 (2.9)	3.3	0.5135	0	Fixed	1.10 (0.70 - 1.72)	0.6766
South Asian (1)	37 (38.5)	22 (22.0)	-	-	-	-	1.75 -	-
Mixed (1)	1 (1.8)	3 (3.8)	-	-	-	-	0.47 -	-
Mideast (2)	37 (18.0)	19 (9.9)	0.1	0.7335	0	Fixed	1.87 (1.10 - 3.18)	0.0208
African (2)	61 (55.5)	26 (23.6)	9.6	0.0019	89.6	Random	3.94 (0.44 -34.95)	0.2187
AC (13)	637 (34.7)	658 (32.9)	60.6	< 0.0001	80.2	Random	1.07 (0.86 - 1.34)	0.5405
Caucasian (1)	90 (39.3)	129 (51.4)	-	-	-	-	0.77 -	-
East Asian (6)	359 (31.6)	360 (27.4)	31.3	< 0.0001	84	Random	1.23 (0.85 - 1.79)	0.2783
South Asian (1)	32 (33.3)	20 (20.0)	-	-	-	-	1.67 -	-
Mixed (1)	21 (36.8)	16 (42.1)	-	-	-	-	0.87 -	-
Mideast (2)	104 (50.5)	91 (47.4)	5.3	0.0208	81.3	Random	1.08 (0.34 - 1.74)	0.7659
African (2)	31 (28.2)	42 (38.2)	7.01	0.0078	85.9	Random	0.54 (0.13 - 2.24)	0.3990
AA (13)	1000 (54.5)	1212 (60.5)	65.9	< 0.0001	81.8	Random	0.88 (0.77 - 1.02)	0.0852
Caucasian (1)	112 (48.9)	97 (38.6)	-	-	-	-	1.27 -	-
East Asian (6)	743 (65.3)	914 (69.7)	26.4	< 0.0001	81.1	Random	0.94 (0.82 - 1.07)	0.3424
South Asian (1)	27 (28.1)	58 (58.0)	-	-	-	-	0.48 -	-
Mixed (1)	35 (61.4)	19 (50.0)	-	-	-	-	1.23 -	-
Mideast (2)	65 (31.6)	82 (42.7)	3.9	0.0495	74.1	Random	0.75 (0.43 - 1.30)	0.3053
African (2)	18 (16.4)	42 (38.2)	2.4	0.1212	58.4	Fixed	0.43 (0.26 - 0.70)	0.0006
CC + AC (13)	835 (45.5)	791 (39.5)	66.9	< 0.0001	82.1	Random	1.20 (1.01 - 1.43)	0.0398
Caucasian (1)	117 (51.1)	154 (61.4)	-	-	-	-	0.83 -	-
East Asian (6)	394 (34.7)	398 (30.3)	29.6	< 0.0001	83.1	Random	1.20 (0.86 - 1.68)	0.2719
South Asian (1)	69 (71.9)	42 (42.0)	-	-	-	-	1.71 -	-
Mixed (1)	22 (38.6)	19 (50.0)	-	-	-	-	0.77 -	-
Mideast (2)	141 (68.4)	110 (57.3)	5.5	0.0188	81.9	Random	1.20 (0.83 - 1.74)	0.3261
African (2)	92 (83.6)	68 (61.8)	1.1	0.2986	7.5	Fixed	1.35 (1.14 - 1.60)	0.0004
**Subgroups**								
CC risk >1 (5 countries)	658 (35.9)	687 (34.3)						
CC (7)	164 (24.9)	92 (13.4)	12.0	0.0626	49.9	Fixed	1.82 (1.45 - 2.29)	< 0.0001
AC (7)	259 (39.4)	301 (43.8)	27.0	0.0001	77.8	Random	0.89 (0.64 - 1.23)	0.4711
AA (7)	235 (35.7)	294 (42.8)	45.4	< 0.0001	86.8	Random	0.75 (0.49 - 1.15)	0.1936
CC + AC (7)	423 (64.3)	393 (57.2)	39.5	< 0.0001	84.8	Random	1.19 (0.93 - 1.51)	0.1658
CC risk <1 (1 country)	57 (3.1)	38 (1.9)						
CC (1)	1 (1.8)	3 (7.9)	-	-	-	-	0.23 -	-
AC (1)	21 (36.8)	16 (42.1)	-	-	-	-	0.87 -	-
AA (1)	35 (61.4)	19 (50.0)	-	-	-	-	1.23 -	-
CC+AC (1)	22 (38.6)	19 (50.0)	-	-	-	-	0.77 -	-
CC risk vary (1 country)	1120 (61.0)	1278 (63.8)						
CC (5)	33 (2.9)	38 (3.0)	1.3	0.7208	0	Fixed	1.02 (0.65 -1.62)	0.9290
AC (5)	357 (31.9)	341 (26.7)	25.2	< 0.0001	84.1	Random	1.37 (0.96 - 1.95)	0.0844
AA (5)	730 (65.2)	899 (70.3)	19.4	0.0007	79.4	Random	0.90 (0.80 - 1.02)	0.0964
CC + AC (5)	390 (34.8)	379 (29.7)	25.0	< 0.0001	84	Random	1.32 (0.95 - 1.84)	0.0959

Note. Q: Cochran's Q; CI: confidence interval. CC risk >1 (5 countries): Netherlands, Taiwan, India, Turkey (2 studies), and Egypt (2 studies); CC risk <1 (1 country): Brazil; CC risk vary (1 country): China (5 studies).

Supplementary Table 5c. Pooled analysis: *MTHFR* 1298 genotype and the risk of congenital heart disease for parents (8 studies).

Genotype by ethnicity (number of studies)	Case (N= 919) n (%)	Control (N= 1416) n (%)	Test of Heterogeneity Q	*p*	I2 %	Statistical Model	Test of Association Risk Ratio (95% CI)	*p*
**CC (8)**	135 (14.7)	154 (10.9)	34.4	< 0.0001	79.6	Random	1.44 (0.78 - 2.66)	0.2411
Caucasian (4)	53 (8.7)	123 (11.6)	3.6	0.3112	16.1	Fixed	0.71 (0.51 - 0.98)	0.0350
East Asian (1)	19 (12.4)	6 (2.8)	-	-	-	-	4.43 -	-
Mixed (1)	4 (8.5)	1 (3.8)	-	-	-	-	2.24 -	-
African (2)	59 (53.6)	24 (21.8)	9.2	0.0025	89.1	Random	3.99 (0.47 - 33.88)	0.2042
**AC (8)**	351 (38.2)	567 (40.0)	12.7	0.0807	44.7	Fixed	0.95 (0.85 - 1.06)	0.3684
Caucasian (4)	259 (42.5)	465 (43.7)	4.9	0.1806	38.6	Fixed	0.94 (0.84 - 1.07)	0.3439
East Asian (1)	39 (25.5)	53 (24.5)	-	-	-	-	1.04 -	-
Mixed (1)	17 (36.2)	10 (38.5)	-	-	-	-	0.94 -	-
African (2)	36 (32.7)	39 (35.5)	7.7	0.0055	87	Random	0.65 (0.15 - 2.86)	0.5658
**AA (8)**	433 (47.1)	695 (49.1)	42.8	< 0.0001	83.7	Random	0.93 (0.74 - 1.18)	0.5733
Caucasian (4)	297 (48.8)	476 (44.7)	12.4	0.006	75.9	Random	1.17 (0.93 - 1.45)	0.1756
East Asian (1)	95 (62.1)	157 (72.7)	-	-	-	-	0.84 -	-
Mixed (1)	26 (55.3)	15 (57.7)	-	-	-	-	0.96 -	-
African (2)	15 (13.6)	47 (42.7)	1.8	0.1774	45	Fixed	0.32 (0.19 - 0.54)	< 0.0001
**CC + AC (8)**	486 (52.9)	721 (50.9)	38.7	< 0.0001	81.9	Random	1.09 (0.88 - 1.34)	0.4291
Caucasian (4)	312 (51.2)	588 (55.3)	10.0	0.0185	70	Random	0.89 (0.73 - 1.08)	0.2333
East Asian (1)	58 (37.9)	59 (27.3)	-	-	-	-	1.39 -	-
Mixed (1)	21 (44.7)	11 (42.3)	-	-	-	-	1.06 -	-
African (2)	95 (86.4)	63 (57.3)	1.0	0.3211	0	Fixed	1.51 (1.26 - 1.80)	< 0.0001
**Subgroups**								
**CC risk >1 (4 countries)**	641 (45.6)	784 (36.7)						
CC (5)	90 (21.8)	44 (8.0)	13.5	0.0092	70.3	Random	2.69 (1.24 - 5.82)	0.0124
AC (5)	138 (33.4)	188 (34.1)	7.6	0.1062	47.6	Fixed	1.00 (0.84 - 1.20)	0.9888
AA (5)	185 (44.8)	320 (58.0)	15.6	0.0036	74.3	Random	0.73 (0.53 - 1.01)	0.0538
CC + AC (5)	228 (55.2)	232 (42.0)	7.4	0.1167	45.9	Fixed	1.29 (1.13 - 1.47)	0.0001
**CC risk <1 (2 countries)**	764 (54.4)	1353 (63.3)						
CC (3)	45 (8.9)	110 (12.7)	2.0	0.3628	1.4	Fixed	0.65 (0.45 - 0.92)	0.0158
AC (3)	213 (42.1)	379 (43.9)	4.4	0.111	54.5	Fixed	0.92 (0.80 - 1.06)	0.2366
AA (3)	248 (49.0)	375 (43.4)	8.9	0.012	77.4	Random	1.24 (0.97 - 1.60)	0.0920
CC + AC (3)	258 (51.0)	489 (56.6)	7.9	0.0191	74.7	Random	0.83 (0.65 - 1.06)	0.1406

Note. Q: Cochran's Q; CI: confidence interval. CC risk >1 (4 countries): Italy, Brazil, China, Egypt (2 studies); CC risk <1 (2 countries): Russia, Netherlands (2 studies)

Supplementary Table 5d. Pooled analysis: *MTHFR* 1298 genotype and the risk of congenital heart disease for mothers (7 studies).

Genotype by ethnicity (number of studies)	Case (N = 691) n (%)	Control (N = 1165) n (%)	Test of Heterogeneity			Statistical Model	Test of Association	
			Q	*p*	I^2 %		Risk Ratio (95% Cl)	*p*
CC (7)	116 (16.8)	117 (10.0)	24.7	0.0004	75.7	Random	1.72 (0.89 - 3.31)	0.1042
Caucasian (3)	34 (8.9)	86 (10.6)	2.2	0.3401	7.3	Fixed	0.81 (0.54 - 1.22)	0.3162
East Asian (1)	19 (12.4)	6 (2.8)	-	-	-	-	4.43 -	-
Mixed (1)	4 (8.5)	1 (3.8)	-	-	-	-	2.24 -	-
African (2)	59 (53.6)	24 (21.8)	9.2	0.0025	89.1	Random	3.99 (0.47 -33.9)	0.2042
AC (7)	253 (36.6)	444 (38.1)	11.4	0.0765	47.4	Fixed	0.98 (0.86 - 1.12)	0.7761
Caucasian (3)	161 (42.3)	342 (42.1)	3.8	0.1509	47.1	Fixed	0.98 (0.84 - 1.15)	0.7834
East Asian (1)	39 (25.5)	53 (24.5)	-	-	-	-	1.04 -	-
Mixed (1)	17 (36.2)	10 (38.5)	-	-	-	-	0.94 -	-
African (2)	36 (32.7)	39 (35.3)	7.7	0.0055	87	Random	0.65 (0.15 - 2.86)	0.5658
AA (7)	322 (46.6)	604 (51.8)	35.7	< 0.0001	83.2	Random	0.87 (0.67 - 1.13)	0.3053
Caucasian (3)	186 (48.8)	385 (47.4)	10.4	0.0054	80.9	Random	1.11 (0.83- 1.48)	0.4800
East Asian (1)	95 (62.1)	157 (72.7)	-	-	-	-	0.84 -	-
Mixed (1)	26 (55.3)	15 (57.7)	-	-	-	-	0.96 -	-
African (2)	15 (13.6)	47 (42.7)	1.8	0.1774	45	Fixed	0.32 (0.19 - 0.54)	< 0.0001
CC + AC (7)	369 (53.4)	561 (48.2)	24.3	0.0005	75.3	Random	1.15 (0.93 - 1.42)	0.1923
Caucasian (3)	195 (51.2)	428 (52.6)	6.5	0.0385	69.3	Random	0.92 (0.70 - 1.19)	0.5158
East Asian (1)	58 (37.9)	59 (27.3)	-	-	-	-	1.39 -	-
Mixed (1)	21 (44.7)	11 (42.3)	-	-	-	-	1.06 -	-
African (2)	95 (86.4)	63 (57.3)	1.0	0.3211	0	Fixed	1.51 (1.26 - 1.80)	< 0.0001
**Subgroups**								
CC risk >1 (4 countries)								
CC (5)	90 (21.8)	44 (8.0)	13.5	0.0092	70.3	Random	2.69 (1.24 - 5.82)	0.0124
AC (5)	138 (33.4)	188 (34.1)	7.6	0.1062	47.6	Fixed	1.00 (0.84 - 1.20)	0.9888
AA (5)	185 (44.8)	320 (58.0)	15.6	0.0036	74.3	Random	0.73 (0.53 - 1.01)	0.0538
CC + AC (5)	228 (55.2)	232 (42.0)	7.4	0.1167	45.9	Fixed	1.29 (1.13 - 1.47)	0.0001
CC risk <1 (2 countries)								
CC (2)	26 (9.4)	73 (11.9)	1.3	0.2459	25.7	Fixed	0.72 (0.45 - 1.16)	0.1818
AC (2)	115 (41.4)	256 (41.8)	3.7	0.0538	73.1	Fixed	0.96 (0.79 - 1.16)	0.6619
AA (2)	137 (49.3)	284 (46.3)	8.2	0.0042	87.8	Random	1.20 (0.79 - 1.83)	0.3957
CC + AC (2)	141 (50.7)	329 (53.7)	6.0	0.0143	83.3	Random	0.80 (0.46 - 1.40)	0.4290

Note. Q: Cochran's Q; CI: confidence interval. CC risk >1 (4 countries): Italy, Brazil, China, and Egypt (2 studies); CC risk <1 (2 countries): Russia and Netherlands.

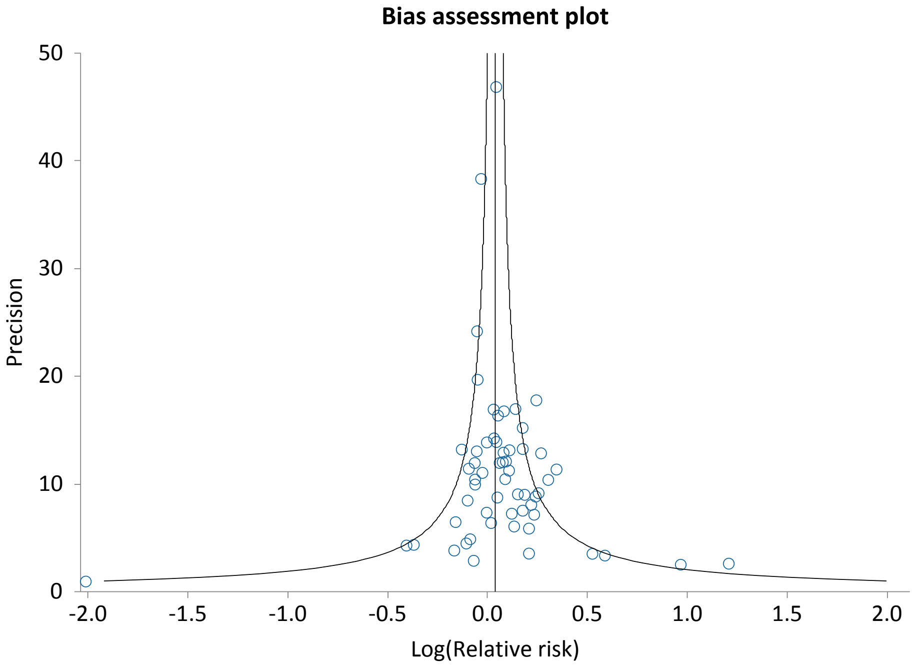

Supplementary Figure 1. The Funnel plot of *MTHFR* 677 TT+CT

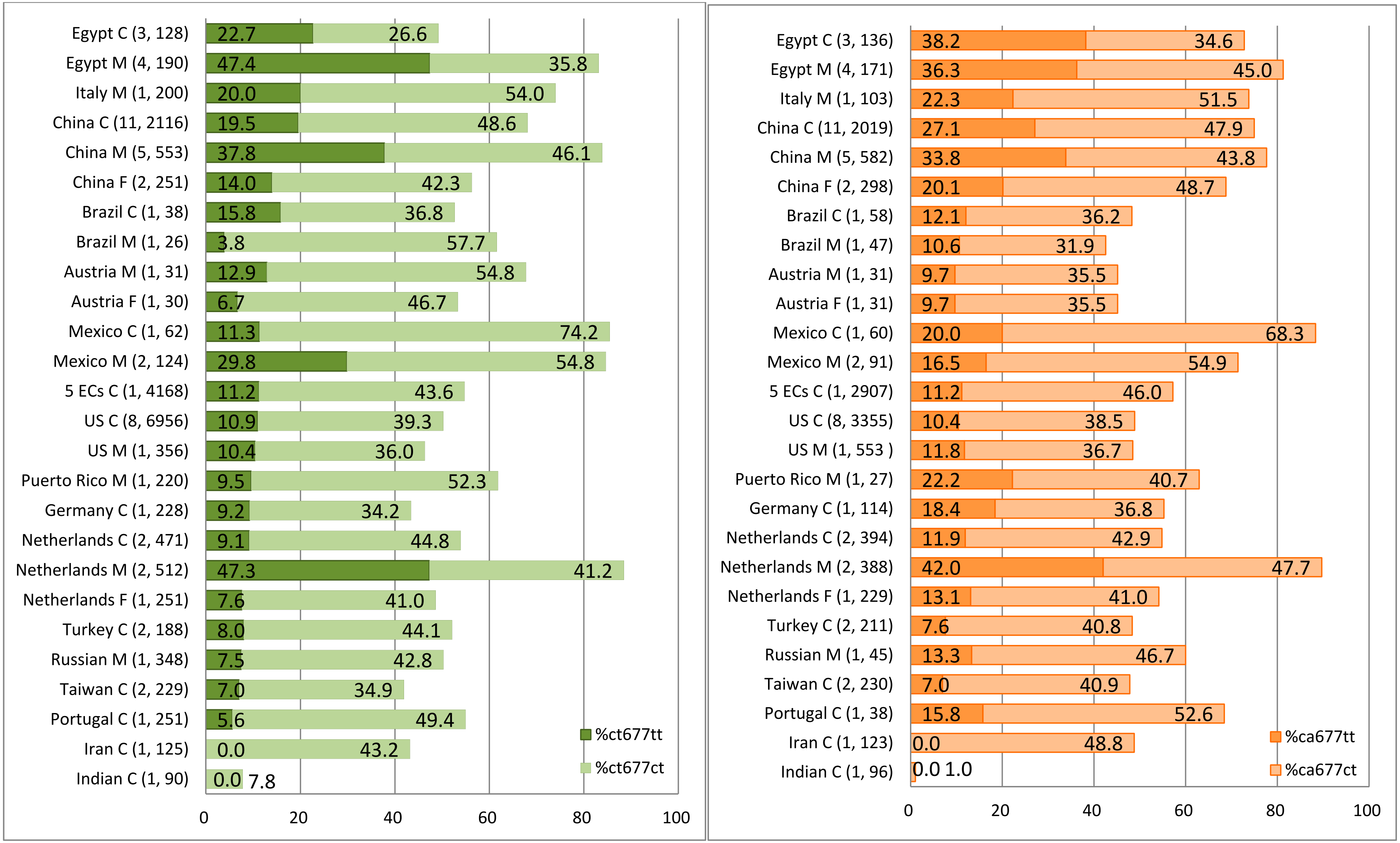

C = children; M = mothers; F = fathers; 5ECs: 5 European Countries: Australia, Belgium, Germany, Netherlands, and United Kingdom.

Supplementary Figure 2. The percent of *MTHFR* 677 TT and CT polymorphism per control (left) and congenital heart disease case (right) groups

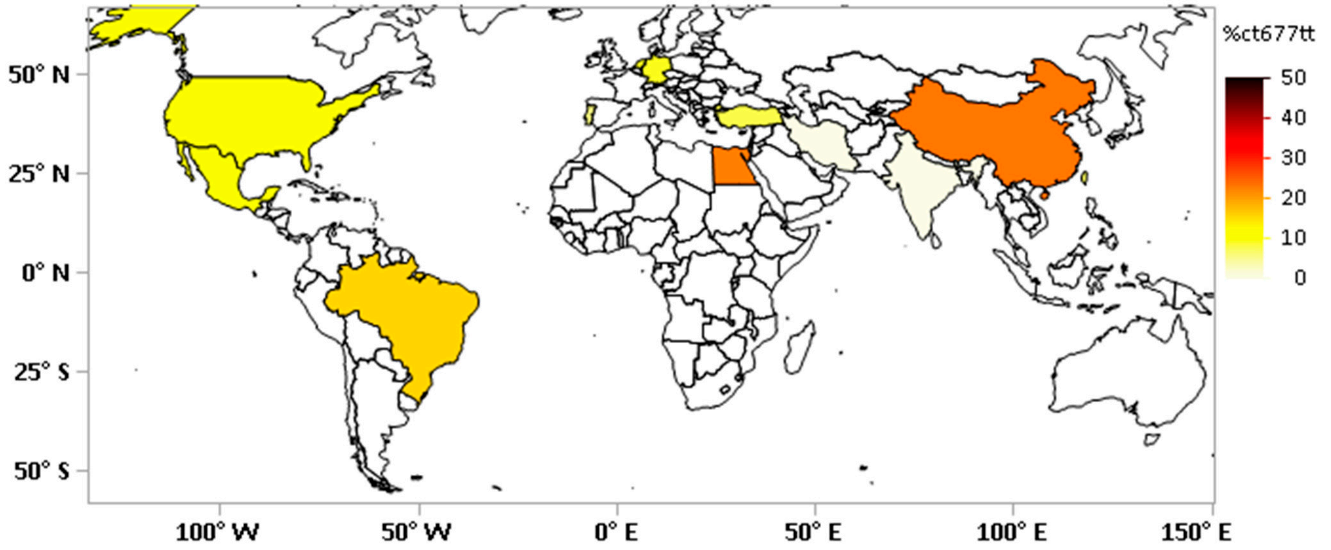

(a)

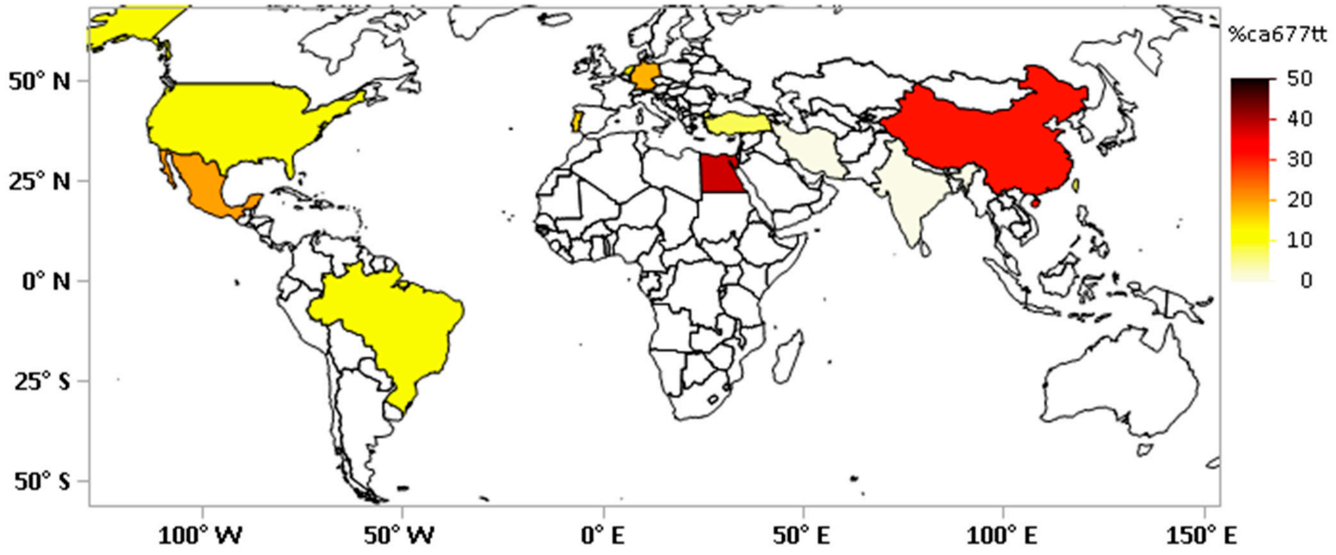

(b)

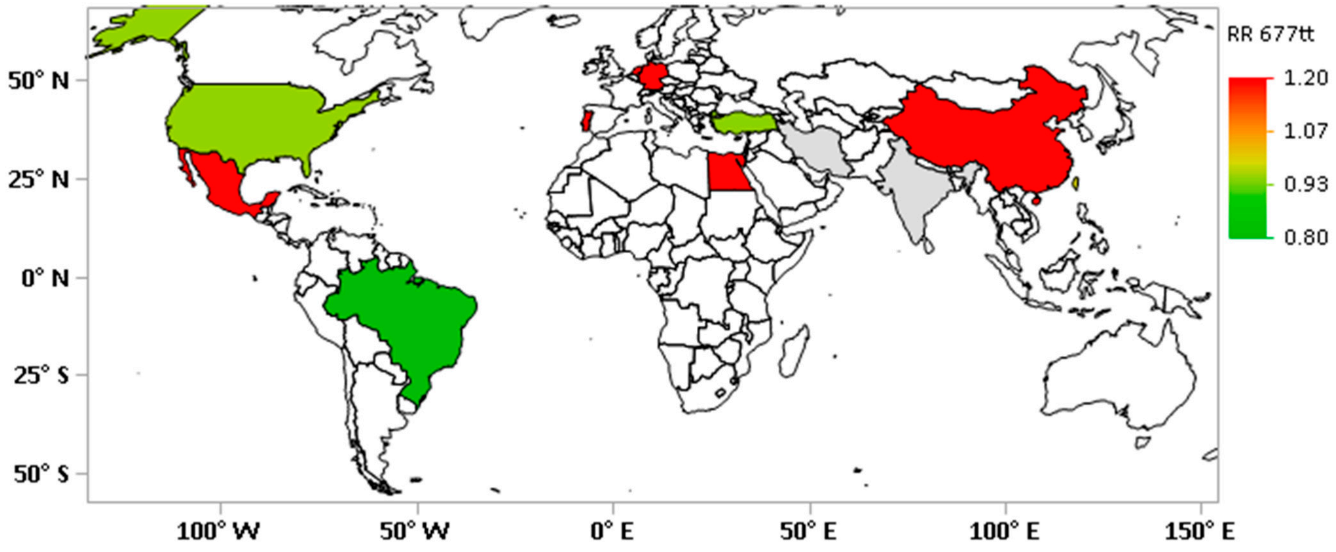

(c)

Supplementary Figure 3. Geographic information maps for the percent of *MTHFR* 677 TT polymorphism (a) per control group (ct), (b) per congenital heart disease (CHD) group (ca) and (c) CHD risk for children.

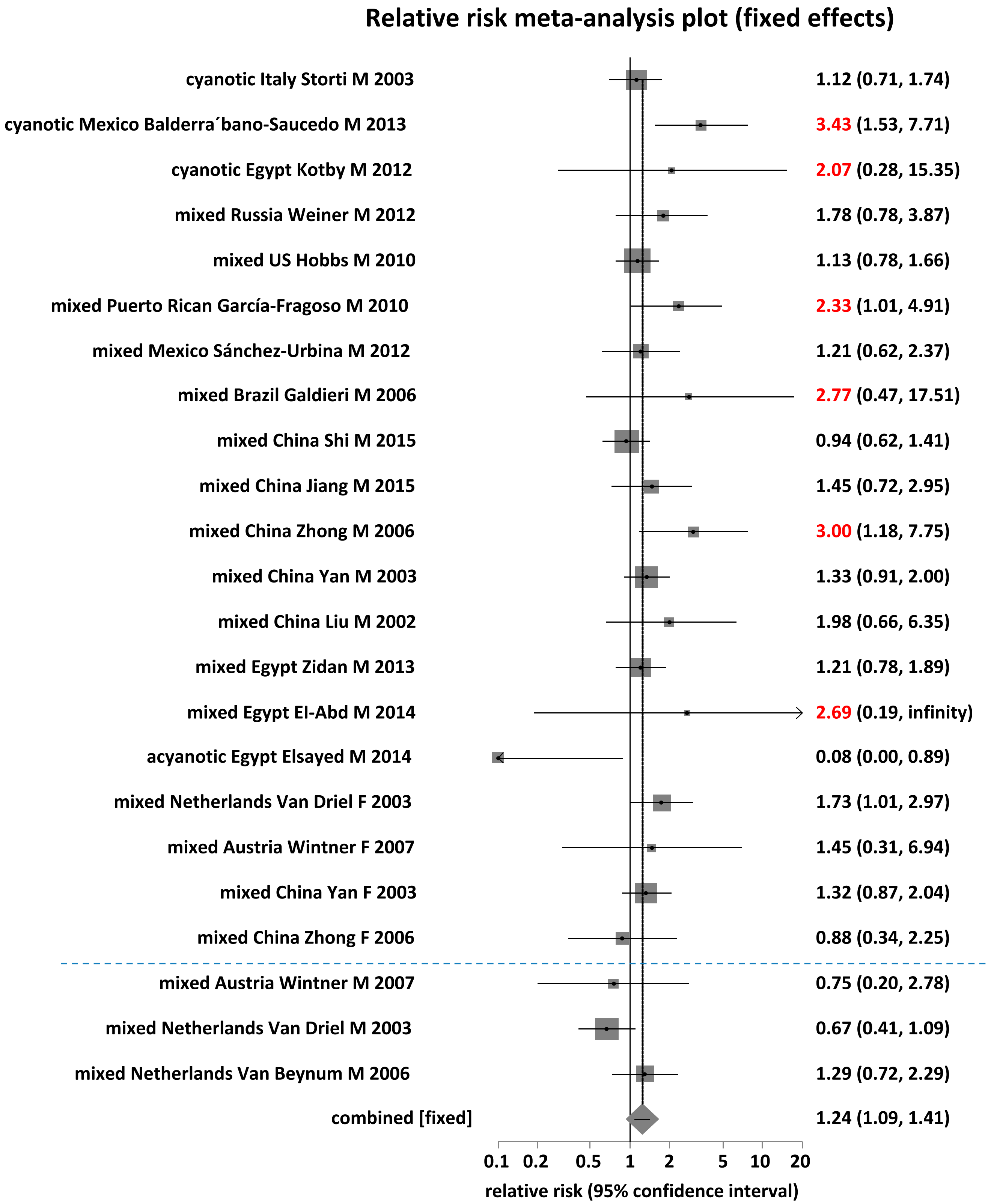

Supplementary Figure 4. Forest plot of risks of congenital heart disease (CHD) by *MTHFR* 677 TT polymorphisms by types of CHD for mothers and fathers.

Note. Cyanotic: cyanotic CHDs; mixed: mixed cyanotic and acyanotic CHDs; acyanotic: acyanotic CHDs; M: mothers; F: fathers; studies above dotted line: TT risk >1; studies under dotted line: TT risk <1; red words: relative risk >2.

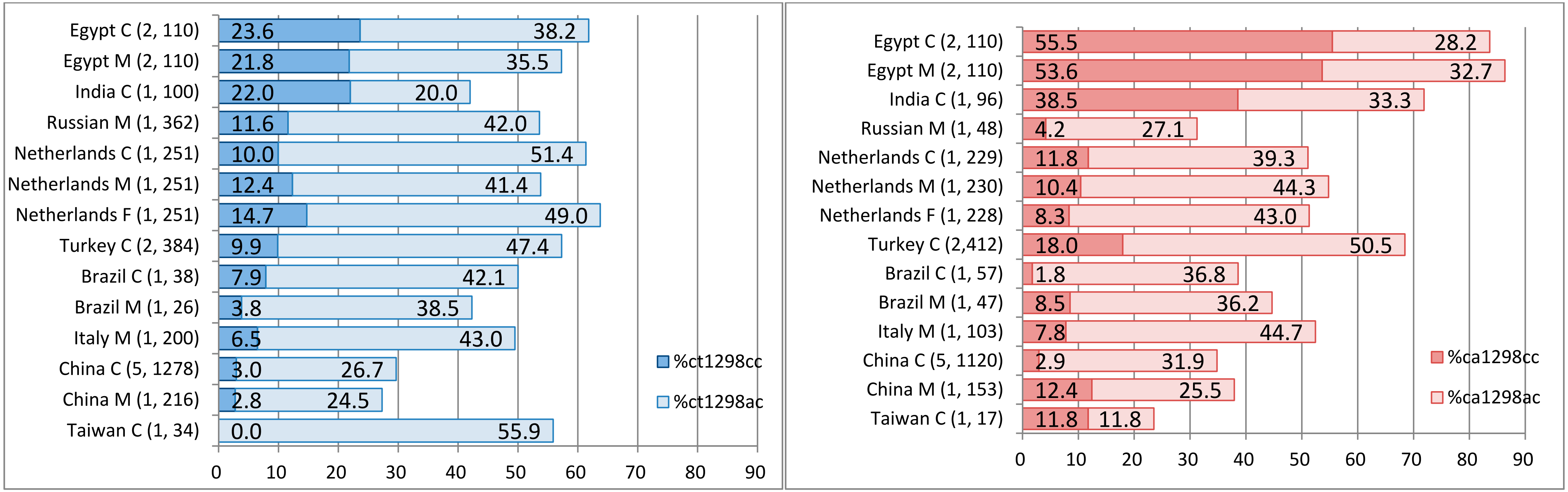

C = children; M = mothers; F = fathers

Supplementary Figure 5. The percent of *MTHFR* 1298 CC and AC polymorphism per control (left side) and congenital heart disease case (right side) groups

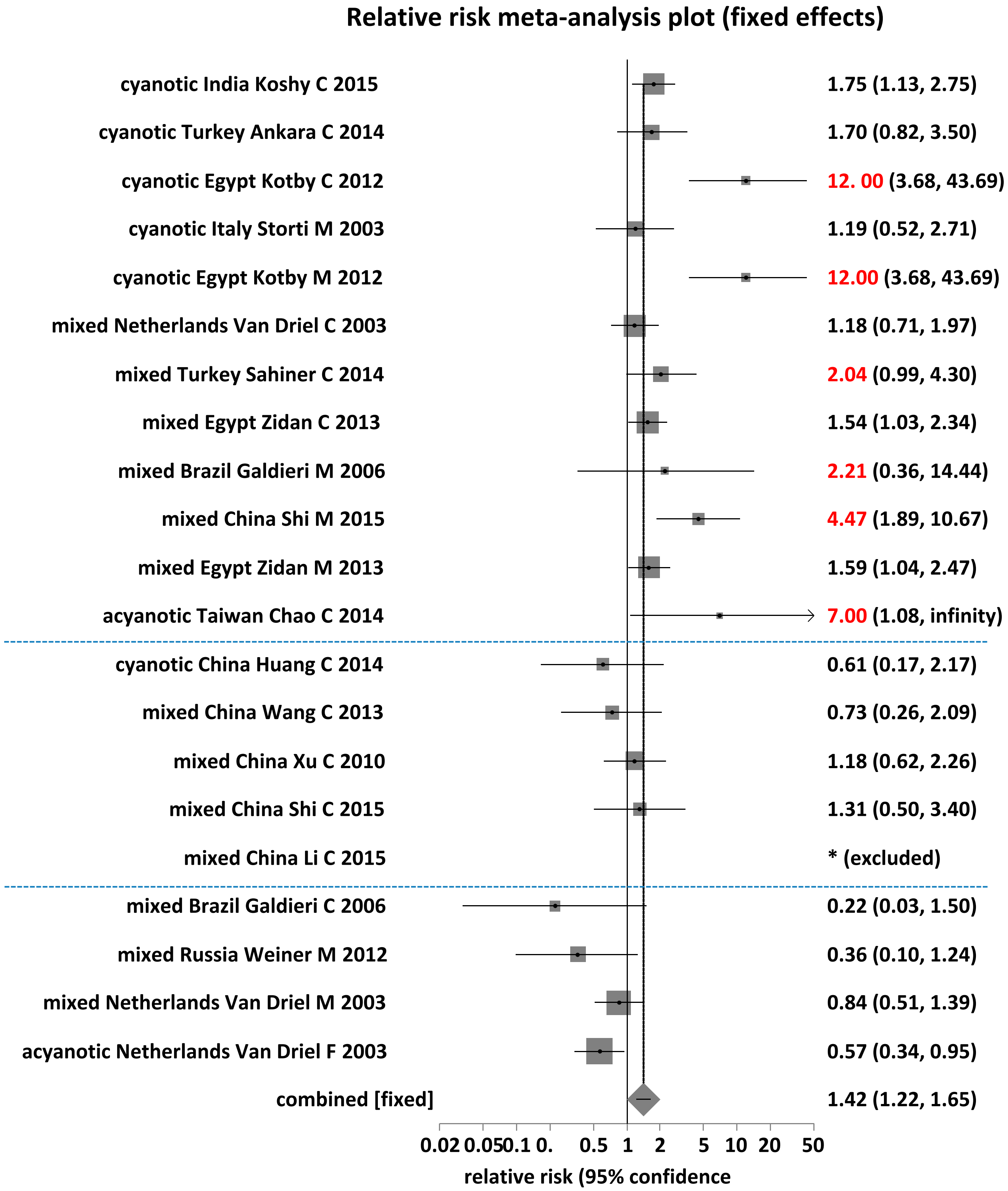

Supplementary Figure 6. Forest plot of risks of congenital heart disease (CHD) by *MTHFR* 1298 CC polymorphisms per countries for children.

Note. Cyanotic: cyanotic CHDs; mixed: mixed cyanotic and acyanotic CHDs; acyanotic: acyanotic CHDs; C: children; M: mother; F: father; studies above dotted line: CC risk >1; studies between dotted lines: CC risk vary; studies under dotted line: CC risk <1; red words: relative risk >2.

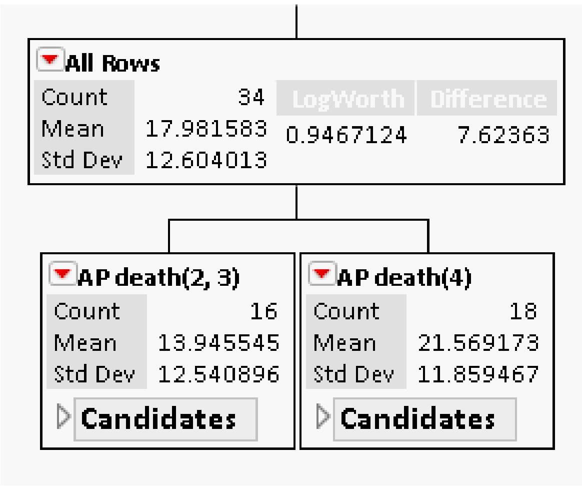

Supplementary Figure 7. Recursive partition tree: the percent of *MTHFR* TT polymorphism by death from air pollution (AP death) for children with congenital heart disease.
